# To Be or Not to Be a Pseudogene: A Molecular Epidemiological Approach to the *mclx* Genes and Its Impact in Tuberculosis

**DOI:** 10.1371/journal.pone.0128983

**Published:** 2015-06-02

**Authors:** Catarina Lopes Santos, Hanna Nebenzahl-Guimaraes, Marta Vaz Mendes, Dick van Soolingen, Margarida Correia-Neves

**Affiliations:** 1 Life and Health Sciences Research Institute (ICVS), School of Health Sciences, University of Minho, Braga, Portugal; 2 ICVS/3B’s, PT Government Associate Laboratory, Braga/Guimarães, Portugal; 3 National Institute for Public Health and the Environment (RIVM), Bilthoven, Netherlands; 4 IBMC—Instituto de Biologia Molecular e Celular, Universidade do Porto, Porto, Portugal; 5 Department of Medical Microbiology, Radboud University Nijmegen Medical Centre, Nijmegen, Netherlands; St. Petersburg Pasteur Institute, RUSSIAN FEDERATION

## Abstract

Tuberculosis presents a myriad of symptoms, progression routes and propagation patterns not yet fully understood. Whereas for a long time research has focused solely on the patient immunity and overall susceptibility, it is nowadays widely accepted that the genetic diversity of its causative agent, *Mycobacterium tuberculosis*, plays a key role in this dynamic. This study focuses on a particular family of genes, the *mclx*s (*Mycobacterium* cyclase/LuxR-like genes), which codify for a particular and nearly mycobacterial-exclusive combination of protein domains. *mclx*s genes were found to be pseudogenized by frameshift-causing insertion(s)/deletion(s) in a considerable number of *M*. *tuberculosis* complex strains and clinical isolates. To discern the functional implications of the pseudogenization, we have analysed the pattern of frameshift-causing mutations in a group of *M*. *tuberculosis* isolates while taking into account their microbial-, patient- and disease-related traits. Our logistic regression-based analyses have revealed disparate effects associated with the transcriptional inactivation of two *mclx* genes. In fact, *mclx2* (Rv1358) pseudogenization appears to be primarily driven by the microbial phylogenetic background, being mainly related to the Euro-American (EAm) lineage; on the other hand, *mclx3* (Rv2488c) presents a higher tendency for pseudogenization among isolates from patients born on the Western Pacific area, and from isolates causing extra-pulmonary infections. These results contribute to the overall knowledge on the biology of *M*. *tuberculosis* infection, whereas at the same time launch the necessary basis for the functional assessment of these so far overlooked genes.

## Introduction

Tuberculosis (TB) is an air-borne contagious disease that remains responsible for high rates of morbidity and mortality worldwide: it is estimated that in 2013, nine million people fell ill with TB and 1.5 million died from it [1]. TB's rate of incidence is declining slowly (1.5% per year in average between 2000 and 2013) [1], which is somehow counter-intuitive given the financial effort put into research and prevention frameworks aiming towards its eradication. The reasons for this halting TB twilight, besides those related with health policies, are the still missing links in the understanding of the disease establishment on its latent or active form and TB transmission. TB has many different facets—from a life-lasting silent infection to an active and potentially deathly disease—and classically most of this variability has been attributed to the hosts’ immune competence. However, and more recently, the role of the etiological agent—*Mycobacterium tuberculosis* (Mtb)—genotype has been gaining more relevance, as several studies came up demonstrating that small genetic variations in clinical isolates or laboratory strains have a significant impact not only in strict microbial characteristics, such as antibiotic resistance (for instance, [2]), but also in factors related with the host-microorganism relationship dynamics, such as disease progression and/or ability to modulate the host's immune response ([3] and references therein).

This work presented here is focused on a family of genes that is almost exclusive of *Mycobacterium* spp. and particularly abundant in members of the *M*. *tuberculosis* complex. Although they have been seldom addressed from a functional point of view, their structure suggests they might be involved in transcriptional regulation and response to quorum sensing (*sensu lato*) stimuli (i.e., communication within bacterial cells or between bacteria and their hosts) [4]. Its uniqueness relies on codifying for a particular combination of domains: an N-terminal CHD (cyclase homology domain) and a C-terminal LuxR HTH (helix turn helix) domain; depending on the domain identification algorithm, an AAA (ATPases associated with several cellular activities)/NB-ARC domain may also be identified between the other two [4–6]. Whereas the AAA/NB-ARC domain has the general function of binding and hydrolysing ATP and/or participating in the protein oligomerization ([5] and references therein), the CHD is the catalytic domain of the class III nucleotidyl (adenylyl/guanylyl) cyclases [5,6], and the LuxR is a DNA-binding domain mostly (but not exclusively) known to be associated with quorum sensing transcription modulation [4,7,8]. In the genome of the reference strain Mtb H37Rv, one can find three genes that codify for proteins with this particular domain composition: Rv0386, Rv1358 and Rv2488c [4–6]. For practical reasons, these genes will be referred to as *mclx1*, *mclx2* and *mclx3* (from *M* *ycobacterium* cyclase/LuxR-like genes), respectively, in the remainder of this manuscript.

Taking into account the overall lack of physiological and functional studies on Mclx proteins, they can either be viewed as putative cyclase proteins with additional non-cyclase domain(s) attached [5,6], or as putative transcriptional regulators from the LuxR family that may respond to, or be modulated by, ATP or cAMP [4]. Interestingly, the genome of the reference strain Mtb H37Rv codifies for 16 genes with a CHD domain, some of which are predicted to be transmembranar and others (including those from the Mclx family) predicted to be soluble [5,6]. Both their frequency and their unique diversity in terms of domains composition suggest that the cyclase activity may be a key point in Mtb fitness. In order to complete their function—bind ATP (or, less commonly, GTP) and convert it to the secondary signal cAMP (or cGMP)—nucleotidyl cyclases require a series of conserved residues that have been previously characterized and that are responsible for binding a divalent metal, for stabilizing the transition state species and for selecting and/or attaching the substrate (either ATP or GTP) [5,6]. Interestingly, only the Mclx1 has all the necessary residues for cyclase activity, as Mclx2 lacks one of the residues necessary for the metal binding and a transition-stabilizing asparagine, Mclx3 lacks a transition-stabilizing asparagine. Moreover, both Mclx2 and Mclx3 seem to lack the substrate selectivity residues [5]. Additionally, the Mclx1 is the only family member that has been functionally characterized: not only was this protein found to have a significant (20%) guanylyl cyclase side activity, besides its adenylyl activity [9], but was also found to have a role in virulence [10]. In fact, Mclx1 was found to be required for a cAMP burst in macrophages upon infection that destabilizes the macrophage immune response: loss of Mclx1 resulted in a reduction in the production of tumor necrosis factor (TNF), and a decrease in the bacterial survival and in the immunopathology in the animal tissues [10].

The general association between the LuxR domain and quorum-sensing mechanisms, together with the lack of some canonical residues for the cyclase activity in the Mclx2 and Mclx3, suggest that these proteins may indeed be quorum-sensing-like transcriptional regulators that respond to or bind to ATP and/or cAMP in an allosteric modulatory fashion. A possible relation between cyclic nucleotides and quorum-sensing (*sensu lato*) is not new: a dynamic relationship between cyclic nucleotides as signals and quorum-sensing regulatory mechanisms has been observed before, either directly or through CRP (cAMP-binding proteins), in organisms such as *Vibrio vulnificus* [11], *Vibrio fischeri* [12], and *Vibrio cholerae* [13], among others.

The study presented here describes an integrative analysis combining genomics and epidemiology and is focused on the clinical consequences of *mclx* variation. Interestingly, we have found a scattered pattern of pseudogenization among *mlcx2* and *mclx3* genes, with implications at the level of patients' demographic characteristics and TB clinical manifestations. As so, this report establishes a link between the functionality of the proteins encoded by these two genes and the virulence-related fitness and host adaptation ability of the Mtb.

## Materials and Methods

### Screening and alignment of the *mclx* genes from public available genomes

The presence and transcriptional integrity of the *mclx* genes was analysed in a panel of Mtb complex strains and clinical isolates which genome had been completely sequenced. These organisms were selected from the Genome database of the National Center for Biotechnology Information (NCBI), limiting the search by organism—*Mycobacterium tuberculosis* complex (taxid: 77643)—and including only genomes with the status "Complete" or "Scaffolds or contigs". This search was performed on the 20^th^ of March 2014 and yielded 187 organisms. To retrieve the *mclx* gene sequences from these organisms, each genome or set of scaffolds was used as a reference against which the *mclx* sequences from the reference strain Mtb H37Rv (Rv0386, Rv1358 and Rv2488c) were mapped, using the assembling tools from Geneious R7.1.4 (Biomatters) [14]. Instances when the putative *mclx* orthologue spanned more than one scaffold/contig lead to the elimination of the respective organism from the analyses, as to avoid sequencing misreads potentially attributed to scaffolds/contigs junctions. One hundred and fifty strains/clinical isolates were retained for further analyses: two of *Mycobacterium africanum*, 12 of *Mycobacterium bovis*, eight of *Mycobacterium cannetti*, and 128 of Mtb (Table 1). The *mclx* genes were identified and individually aligned against their reference orthologue from Mtb H37Rv to identify nucleotide substitutions, insertions and deletions, using the ClustalW algorithm [15] available in the Geneious software [14] (S1A Fig).

**Table 1:** Pairwise identity and indels in the *mclx* genes in a panel of 150 Mtb complex strains/clinical isolates^1^.

Organism	Lineage	*mclx* 1	*mclx* 2	*mclx* 3
Maf GM041182	WA-2	**99.97%—del -1406 (T)**	99.90%	99.90%
Maf K85	WA-2	**99.90%—del -1406 (T); del—2481 C)**	99.90%	99.97%
Mbv 04–303	-	99.97%	99.90%	100.00%
Mbv AF2122/97	-	99.90%	99.90%	100.00%
Mbv AN5	-	100.00%	99.90%	100.00%
Mbv BCG ATCC 35733	-	100.00%	99.90%	99.97%
Mbv BCG ATCC 35740	-	99.90%	99.90%	99.97%
Mbv BCG China	-	100.00%	99.90%	**99.80%—ins—2198–2202 (GGCGG)**
Mbv BCG Frappier	-	100.00%	99.90%	99.97%
Mbv BCG Korea 1168P	-	100.00%	99.90%	99.97%
Mbv BCG Mexico	-	100.00%	99.90%	99.97%
Mbv BCG Moreau	-	100.00%	99.90%	99.97%
Mbv BCG Pasteur 1173P2	-	100.00%	99.90%	99.90%
Mbv BCG Tokyo 172	-	100.00%	99.90%	99.97%
Mcn CIPT 140010059	-	99.90%	99.90%	93.40%—ins—2519–2521 (CCA)
Mcn CIPT 140060008	-	99.80%	99.60%	93.50%—ins—2521–2523 (ACC)
Mcn CIPT 140070002	-	99.90%	99.90%	98.90%
Mcn CIPT 140070005	-	99.70%	99.60%	98.60%
Mcn CIPT 140070007	-	99.30%	99.50%	99.00%
Mcn CIPT 140070008	-	99.70%	99.90%	99.10%
Mcn CIPT 140070013	-	99.30%	98.50%	98.80%
Mcn CIPT 140070017	-	99.00%	99.20%	97.00%—ins—2531–2533 (GCC)
Mtb '98-R604 INH-RIF-EM'	EAm	100.00%	**99.90%—ins—840 (T)**	99.97%—del—2716 (A)
Mtb 02_1987	EAs	99.90%	99.97%	100.00%
Mtb 1034	EAs	99.97%	99.97%	100.00%
Mtb 210	EAs	99.97%	99.97%	99.97%
Mtb 43–16836	IO	100.00%	99.97%	**99.90%—ins—1393–1394 (TA)**
Mtb 7199–99	EAm	100.00%	99.90%	100.00%
Mtb BT1	EAs	99.97%	99.97%	99.97%
Mtb BT2	EAs	99.97%	99.97%	99.97%
Mtb BTB05-552	EAm	100.00%	99.97%	100.00%
Mtb BTB05-559	EAm	100.00%	99.97%	100.00%
Mtb C	EAm	99.50%—ins—3195 (T); ins—3211 (G); ins—3218 (G)	99.90%	99.90%—ins—2919 (G); ins—2992 (G)
Mtb CAS/NITR204	EAI	**98.90%- 15 del/ 1 ins**	99.20%- 13 del	**98.70%- 19 del/ 2 ins**
Mtb CCDC5079	EAs	99.97%	99.90%—del—2523–2525 (CGA)	99.97%
Mtb CCDC5180	EAs	99.97%	99.97%	99.97%
Mtb CDC1551	EAm	100.00%	99.96%	100.00%
Mtb CDC1551A		100.00%	99.97%	100.00%
Mtb CTRI-2	EAm	100.00%	**99.90%—ins—840 (T)**	100.00%
Mtb EAI5		100.00%	99.90%	**99.90%—del—1388–1392 (TTGCG)**
Mtb EAI5/NITR206	IO	99.90%	99.97%	100.00%
Mtb EAS054	IO	99.97%	99.97%	**99.80%—del—1388–1392 (TTGCG)**
Mtb F11	EAm	99.97%	**99.90%—ins—840 (T)**	100.00%
Mtb FJ05194	EAs	100.00%	99.90%	100.00%
Mtb GuangZ0019	EAm	100.00%	99.97%	100.00%
Mtb H37Ra	EAm	100.00%	100.00%	100.00%
Mtb H37RvCO	EAm	100.00%	100.00%	100.00%
Mtb HKBS1	EAs	99.97%	99.97%	99.97%
Mtb HM	EAm*	99.97%	**99.90%—ins—840 (T)**	99.00%—ins—1921–1953 (TG. . .TG)
Mtb HN878	EAs	99.97%	99.97%	99.97%
Mtb INS_MDR	EAm	100.00%	99.97%	100.00%
Mtb INS_SEN	EAm	100.00%	99.97%	100.00%
Mtb INS_XDR	EAm	100.00%	99.97%	100.00%
Mtb KZN 1435	EAm	100.00%	**99.90%—ins—840 (T)**	99.97%
Mtb KZN 4207	EAm	100.00%	**99.90%—ins—840 (T)**	99.97%
Mtb KZN 605	EAm	100.00%	**99.90%—ins—840 (T)**	99.97%
Mtb KZN R506	EAm	100.00%	**99.90%—ins—840 (T)**	99.97%
Mtb KZN V2475	EAm	100.00%	**99.90%—ins—840 (T)**	99.97%
Mtb MTB-489	?	99.97%	100.00%	99.97%
Mtb NA-A0008	?	**99.97%—del—871 (C)**	99.90%	**99.80%—del—1388–1392 (TTGCG); del—2044 (C)**
Mtb NA-A0009	?	**99.90%—del—871 (C); ins—2308–2310 (AG); del—2901 (C)**	99.97%	**99.80%—del—1388–1392 (TTGCG); del—2104 (G)**
Mtb NCGM2209	EAs	99.97%	99.97%	100.00%
Mtb OSDD071	EAI	100.00%	99.97%	99.90%
Mtb OSDD105	EAm	100.00%	99.90%	**99.97%—del—900 (C)**
Mtb OSDD493	EAm	**99.97%—del—352 (C)**	99.97%	100.00%
Mtb PanR0201	EAm	100.00%	**99.90%—ins—840 (T)**	100.00%
Mtb PanR0202	EAm	100.00%	99.97%	100.00%
Mtb PanR0205	EAm	100.00%	**99.90%—ins—840 (T)**	99.97%
Mtb PanR0206	EAm	100.00%	99.90%	100.00%
Mtb PanR0207	EAm	100.00%	**99.90%—ins—840 (T)**	99.97%
Mtb PanR0208	EAm	100.00%	99.97%	100.00%
Mtb PanR0209	EAm	100.00%	**99.90%—ins—840 (T)**	99.97%
Mtb PanR0304	EAm	100.00%	**99.90%—ins—840 (T)**	100.00%
Mtb PanR0305	EAm	100.00%	99.97%	100.00%
Mtb PanR0306	EAm	100.00%	**99.90%—ins—840 (T)**	99.97%
Mtb PanR0307	EAm	100.00%	**99.90%—ins—840 (T)**	99.97%
Mtb PanR0308	EAm	100.00%	**99.90%—ins—840 (T)**	100.00%
Mtb PanR0309	EAm	100.00%	99.97%	100.00%
Mtb PanR0313	EAm	100.00%	**99.90%—ins—840 (T)**	100.00%
Mtb PanR0314	EAm	100.00%	**99.90%—ins—840 (T)**	99.97%
Mtb PanR0315	EAm	100.00%	99.90%	100.00%
Mtb PanR0316	EAm	100.00%	99.97%	99.97%
Mtb PanR0317	EAm	100.00%	**99.90%—ins—840 (T)**	99.97%
Mtb PanR0401	EAm	100.00%	**99.90%—ins—840 (T)**	99.97%
Mtb PanR0402	EAm	100.00%	**99.10%—ins—840 (T); del—3466 (G)**	99.97%
Mtb PanR0403	EAm	100.00%	**99.90%—ins—840 (T)**	100.00%
Mtb PanR0404	EAm	100.00%	**99.90%—ins—840 (T)**	100.00%
Mtb PanR0405	EAm	100.00%	**99.90%—ins—840 (T)**	99.97%
Mtb PanR0409	EAm	100.00%	**99.90%—ins—840 (T)**	100.00%
Mtb PanR0410	EAm	100.00%	**99.90%—ins—840 (T)**	99.97%
Mtb PanR0411	EAm	100.00%	**99.90%—ins—840 (T)**	99.97%
Mtb PanR0412	EAm	100.00%	**99.90%—ins—840 (T)**	99.97%
Mtb PanR0501	EAm	100.00%	**99.90%—ins—840 (T)**	99.97%
Mtb PanR0503	EAm	100.00%	**99.90%—ins—840 (T)**	100.00%
Mtb PanR0505	EAm	100.00%	**99.90%—ins—840 (T)**	99.97%
Mtb PanR0602	EAm	100.00%	**96.50%- 5 del/ 4 ins**	100.00%
Mtb PanR0603	EAm	100.00%	**99.90%-ins—840 (T)**	99.97%
Mtb PanR0604	EAm	100.00%	**99.90%-ins—840 (T)**	99.97%
Mtb PanR0605	EAs	99.97%	99.97%	99.97%
Mtb PanR0606	EAs	99.97%	99.97%	99.97%
Mtb PanR0607	EAm	100.00%	**99.90%—ins—840 (T)**	99.97%
Mtb PanR0609	EAm	100.00%	**99.90%—ins—840 (T)**	99.97%
Mtb PanR0610	EAm	100.00%	**99.90%—ins—840 (T)**	100.00%
Mtb PanR0611	EAm	99.97%	**99.90%—ins—840 (T)**	99.97%
Mtb PanR0702	EAm	100.00%	**99.90%—ins—840 (T)**	99.97%
Mtb PanR0703	EAm	99.97%	**99.90%—ins—840 (T)**	99.97%
Mtb PanR0704	EAm	99.97%	**99.90%—ins—840 (T)**	99.97%
Mtb PanR0707	EAm	100.00%	**99.90%—ins—840 (T)**	99.97%
Mtb PanR0708	EAm	99.97%	**99.90%—ins—840 (T)**	99.97%
Mtb PanR0801	EAm	100.00%	99.90%	100.00%
Mtb PanR0802	EAm	100.00%	**99.90%—ins—840 (T)**	99.97%
Mtb PanR0803	EAm	100.00%	**99.90%—ins—840 (T)**	99.97%
Mtb PanR0804	EAm	100.00%	**99.90%—ins—840 (T)**	99.97%
Mtb PanR0805	EAm	99.97%	**99.90%—ins—840 (T)**	99.97%
Mtb PanR0902	EAm	100.00%	99.90%	100.00%
Mtb PanR0903	EAm	100.00%	**99.90%—ins—840 (T)**	100.00%
Mtb PanR0904	EAm	100.00%	**99.90%—ins—840 (T)**	99.97%
Mtb PanR0906	EAm	99.97%	**99.90%—ins—840 (T)**	99.97%
Mtb PanR0907	EAm	100.00%	99.90%	100.00%
Mtb PanR0909	EAm	100.00%	**99.90%—ins—840 (T)**	99.97%
Mtb PanR1005	EAm	100.00%	**99.90%—ins—840 (T)**	99.97%
Mtb PanR1006	EAm	100.00%	**99.90%—ins—840 (T)**	99.97%
Mtb PanR1007	EAm	99.97%	**99.90%—ins—840 (T)**	99.97%
Mtb PanR1101	EAm	100.00%	**99.90%—ins—840 (T)**	100.00%
Mtb PR05	?	100.00%	99.97%	**99.80%—del—1388–1392 (TTGCG)**
Mtb R1207	EAs	99.90%	99.97%	100.00%
Mtb RGTB327	EAm*	**99.90%—ins—454 (G); ins—684 (A)**	**99.90%—ins—840 (T); ins—1331 (C)**	**99.90%—ins—766 (G); ins—2844–2845 (GG)**
Mtb RGTB423	IO*	99.97%—ins—2821 (C)	**99.90%—ins—390 (A)**	**99.70%—ins—1121 (A); ins—2160–2162 (GCC); ins—3017 (G)**
Mtb S96-129	EAm	100.00%	99.97%	100.00%
Mtb Beijing/NITR203	EAs	99.90%	99.90%	99.97%
Mtb Erdman = ATCC 35801	EAm	100.00%	99.90%	100.00%
Mtb Haarlem	EAm	99.10%- 7 ins	99.90%	**99.90%—del—2264 (G);del—2362 (G)**
Mtb OSDD515	EAm	100.00%	99.97%	**99.97%—del—938 (T)**
Mtb SUMu001	?	**99.97%—del—952 (G)**	100.00%	100.00%
Mtb SUMu002	?	**99.97%—del—952 (G)**	99.97%	99.97%—ins—2487 (A)
Mtb SUMu003	?	100.00%	99.97%	100.00%
Mtb SUMu004	?	100.00%	99.97%	100.00%
Mtb SUMu005	?	100.00%	99.97%	100.00%
Mtb SUMu006	?	100.00%	99.97%	100.00%
Mtb SUMu008	?	**99.97%—del—952 (G)**	99.97%	100.00%
Mtb SUMu010	?	100.00%	100.00%	100.00%
Mtb SUMu011	?	100.00%	100.00%	100.00%
Mtb SUMu012	?	**99.90%—del—2254 (G); del—2962 (G)**	100.00%	100.00%
Mtb UM 1072388579	?	100.00%	99.90%	100.00%
Mtb UT205	EAm	100.00%	**99.90%—ins—840 (T)**	100.00%
Mtb W-148	EAs	99.97%	99.90%—del—2523–2525 (CGA)	99.97%
Mtb WX3	EAs	99.97%	99.97%	99.97%
Mtb X122	EAs	99.97%	99.90%—del—2523–2525 (CGA)	99.97%
Mtb XDR1219	EAs	99.97%	99.97%	99.97%
Mtb XDR1221	EAs	99.90%	99.97%	100.00%

^1^The pairwise identity refers to the % of conserved residues of each gene after aligning it with its orthologue from the reference strain *M*. *tuberculosis* H37Rv; the putative pseudogenes are highlighted in bold; indel ocurrences are described by their type (ins, insertion; del, deletion) and by their location (considering an alignment with the reference mclx genes from the Mtb H37Rv strain), except when the total number of isolated occurences exceeds 3, in which case only their frequency is indicated; Maf, *Mycobacterium africanum*; Mvb, *Mycobacterium bovis*; Mcn, *Mycobacterium cannetti*; Mtb, *Mycobacterium tuberculosis*; WA-2, West-African 2; EAm, Euro-American lineage; EAs, East-Asian lineage; IO, Indo-Oceanic; EAI, East-African Indian lineage

*, indicates that the information on the lineage was obtained by aligning the H37Rv RD with the genome/scaffolds of the respective organism.

For most Mtb strains/clinical isolates, the information on the lineage could be retrieved from the literature and/or information on the genome. For the few cases in which this information could not be found, each genome or set of scaffolds was used as a reference against which the regions of difference (RD) from the reference strain Mtb H37Rv, described by Gagneux *et al*. [16] in the definition of the six phylogeographical lineages, were mapped, and the occurrence of the described long sequence polymorphisms was used for the definition of the lineage (marked with an "*" in Table 1). In a few cases the RDs fell in scaffolds/contigs junctions, and therefore the presence of polymorphisms could not be accurately determined, precluding the determination of the lineage (marked with a "?" in Table 1).

### Screening and alignment of the *mclx* genes from clinical isolates of an epidemiologically characterized cohort

To get an insight into the epidemiological/clinical features that may be associated with the occurrence of the *mclx* pseudogenization, the *mclx* transcriptional integrity was analysed in a diversified panel of Mtb clinical isolates. This panel is composed of 140 organisms collected and isolated in the Netherlands from 1993 to 2011, which are fully characterized from an epidemiological and clinical point of view (S1 Table) [17]. Demographic and clinical information, provided by the Registration Committee of the Netherlands Tuberculosis Register (NTR) that approved this retrospective study, were linked to the isolates on the basis of gender, date of birth, year of diagnosis and postal code. For 100 of these clinical isolates, whole-genome sequencing was performed ([17] and Nebenzahl-Guimaraes *et al*., unpublished data) and the single nucleotide polymorphisms and INDELs (insertions or deletions) were called against the reference strain H37Rv using Breseq software (version 0.23) with a minimum depth of 15x [18] (S1B Fig). SNPs with low-quality evidence (i.e. possible mixed read alignment) or within 5 bp of an INDEL were discarded. The presence of INDELs within the *mclx* coding regions and their potential to disrupt the open reading frame was evaluated. For the remaining 40 clinical isolates, the *mclx* genes were PCR-amplified in 3 overlapping fragments, each of 1200 to 1400 base pairs (see S2 Table for information on the primers and PCR conditions), and the purified PCR products were sequenced (by GATC Biotech). Each gene fragment was amplified twice and all fragments were sequenced in both directions. The final sequences were mapped against and aligned with their orthologues from the Mtb H37Rv reference strain, using the Geneious R7.1.4 (Biomatters) [14] software (S1C Fig). All alignments were visually inspected and a conservative approach was applied: whenever the sequencing results failed to converge to an obvious consensus, the gene status (functional/pseudogene) was considered to be unknown. For that reason, a few clinical isolates were not considered in the final analyses (final *n* = 127).

### Statistical analyses

To envisage the relationship between the clinical and epidemiological features of the TB infection and the status of the *mclx* genes, a separate logistic regression-based analysis was carried out for *mclx2* and *mclx3*. Firstly, simple binary logistic regressions were performed to identify the significant predictors of each *mclx* genes status. Afterwards, two multiple binary logistic regressions were performed considering only those considered to be significant predictor variables (*p*<0.2 in the univariate analysis). Particularly in the case of *mclx3*, a sequential binary regression model was performed considering three sets of variables (patient-related, microorganism-related and disease-related). All statistical analyses were performed using the IBM SPSS Statistics, version 22 (IBM).

### Phylogenetic tree construction

The FASTA files of publicly available NCBI strains were downloaded and pair aligned with the H37Rv reference sequence (NC_000962.gbk) using MAUVE v2.4.0. Multiple sequence alignments (MSA) using 62 robust SNP markers that have been shown to construct high resolution and reproducible phylogenies [19] were then made for 100 of the clinical isolates and 100 of the publicly available NCBI strains. The MSAs were subsequently used to generate a parsimony-based tree using the DNA parsimony algorithm version 3.69 from the Phylip package.

## Results

### A number of *mclx* genes among the *M*. *tuberculosis* complex species are likely pseudogenized

The *mclx* genes were previously identified as a family of genes nearly exclusive to the *Mycobacterium* genus and particularly abundant among the members of Mtb complex [4]. As a distinctive feature, these genes encode proteins with a particular domain architecture, including a CHD (cyclase homology domain) and a LuxR HTH domain. In order to acquire a better understanding of the distribution of this particular group of genes among the Mtb complex, an alignment-based screening was performed against a diversified panel of organisms with their genome fully-sequenced, including elements from the species *M*. *africanum*, *M*. *bovis*, *M*. *cannetti* and Mtb. Interestingly, this strategy revealed that even though *mclx* genes have in general an overall high degree of sequence conservation, usually with more than 99% nucleotides identical to their orthologues from the reference strain Mtb H37Rv, in a number of them a few INDELs were present, which caused a disruption in the open reading frame leading to the accumulation of stop codons and to the truncation of the respective sequence (Table 1). To avoid misinterpretations, and keeping in mind that gene size can vary to a certain extent without necessarily implying loss of function, the following criterion was established: any given *mclx* gene was considered to be a pseudogene whenever its sequence was truncated in more than 25% of the size of the corresponding Mtb H37Rv reference orthologue. While this criterion would require further functional validation, we consider it appropriate for this initial screening, as it likely minimizes the chance of false positives (i.e., genes that could be considered pseudogenes given a small size variation but that in fact retain their full functionality).

**Fig 1:**
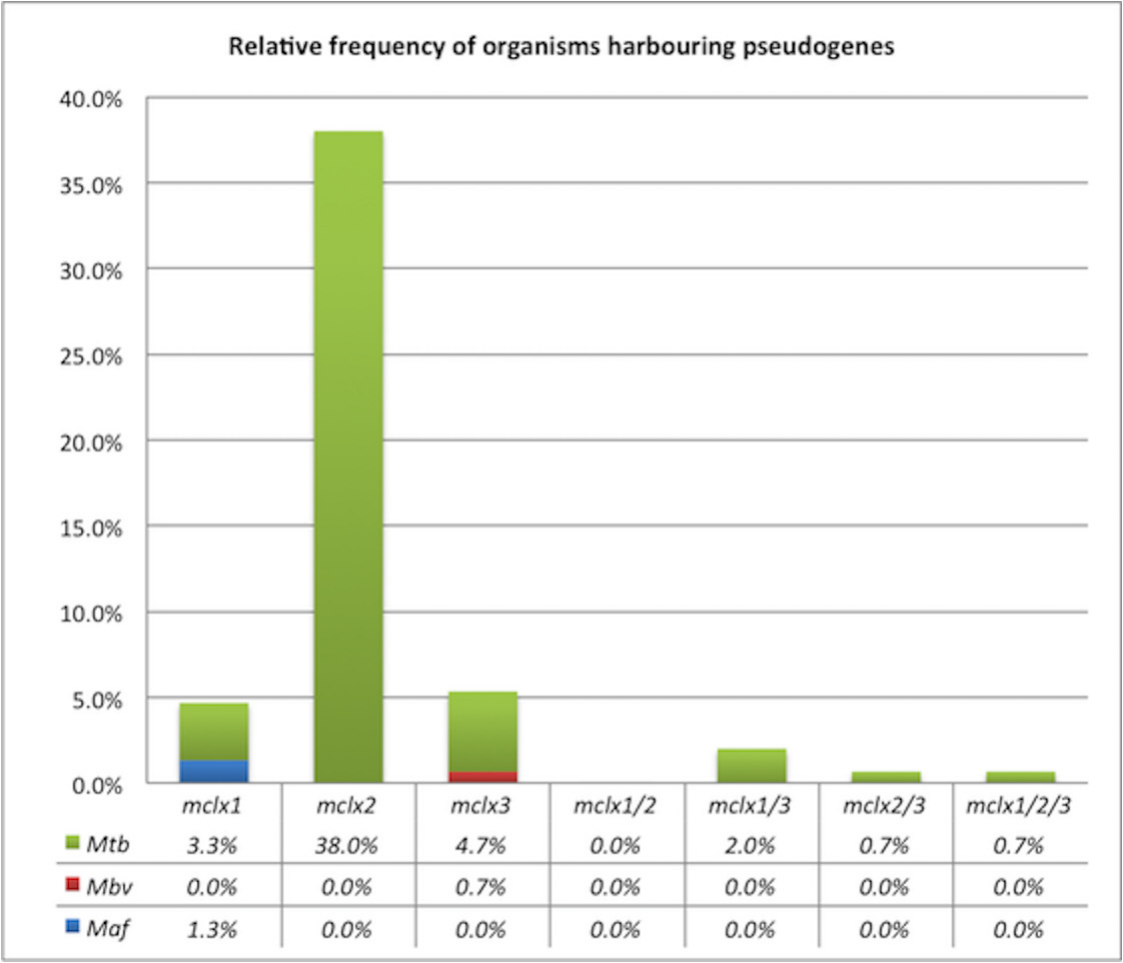
Relative frequency of the organisms harbouring pseudogenes in each of the *mclx* genes. The percentages are relative to the total number of strains/organisms listed in Table 1, and are stratified according to the three main species: Mtb (*Mycobacterium tuberculosis*), Mbv (*Mycobacterium bovis*) and Maf (*Mycobacterium africanum*).

Overall, our analysis revealed that the pseudogenization is a rather common phenomenon among the *mclx* genes: 51.3% of the organisms in the analysed panel have at least one of their *mclx* genes truncated (Table 1 and Fig 1). As shown in Fig 1, the occurrence of the pseudogenes is not evenly distributed among the three different genes: the number of species with a *mclx2* pseudogene is much higher than that of species that suffered pseudogenization in the *mclx1* and/or the *mclx3*. In fact, whereas 7.3% and 8.7% of the organisms in the analysed panel have a pseudogenized *mclx1* and *mclx3*, respectively, 39.3% do so for the *mcx2*. Accordingly, the percentage of organisms with more than 1 *mclx* pseudogenized is relatively low (3.3%). Notwithstanding, it should be highlighted that in three (out of the four) organisms with two pseudogenes, the pseudogenization events occur in the *mclx1* and *mclx*3 (Table 1 and Fig 1), the two genes with lower pseudogenization occurrence, suggesting that the simultaneous loss of *mclx2* and any other *mclx* may be somehow harmful to the microorganisms.

### Epidemiological assessment of the *mclx* pseudogenization

In order to evaluate whether there was a relationship between the inactivation of these genes in certain bacterial isolates and the epidemiological and clinical characteristics of the disease caused by those isolates, the presence and functionality of the *mclx* genes was analysed in a panel of Mtb strains isolated in the Netherlands from 1993 to 2011 and fully characterized regarding their epidemiological and clinical features (S1 and S2 Tables). Even though all organisms in this panel were isolated in the Netherlands, only a minority of them (22.0%) were from native Dutch individuals. In fact, the patients' birth region is quite diversified: whereas 29.9% of the patients were born in Europe, 18.1% were born in Africa, 18.1% in the Eastern Mediterranean area, 13.4% in the South East Asia, 10.2% in the Western Pacific and 9.4% in the Americas. Accordingly, and given the strong phylogeographical nature of the Mtb lineages, this panel of isolates holds representatives of the EAm lineage (51.2%), Indo-Oceanic (IO) lineage (24.4%), East-African Indian (EAI) lineage (14.2%), and East-Asian (EAs) lineage (7.9%). From the classical risk factors commonly associated with active TB, the one that stands out in this panel is the origin from an endemic region. As for the other frequently-mentioned risk factors, their occurrence is rather low: only 3.1% of the strains were known to be isolated from homeless patients, 8.7% from known drug and/or alcohol users, and 14.2% from patients with co-morbidities (10.2% were HIV-positive, 3.1% had diabetes mellitus, 0.8% reported a malignancy and none were diagnosed with renal insufficiency or had been through organ transplantation). Regarding the TB localization, 64.6% of the isolates were retrieved from patients diagnosed with pulmonary TB, 18.9% from patients with extra-pulmonary TB, and 15.7% from patients reported to have both pulmonary and extra-pulmonary TB. Microbial transmissibility was defined following the work of Nebenzahl-Guimaraes *et al*. [17], and 61.4% of the clinical isolates analysed were considered "transmissible". In what concerns the transcriptional integrity of the *mclx* genes, no pseudogenes were found among *mclx1* following the criteria described in the material and methods, whereas 18.9% and 24.4% of the *mclx2* and *mclx3*, respectively, had suffered pseudogenization (S1 Table).

To identify the significant predictors of each *mclx2* and *mclx3* gene status, simple binary logistic regressions were performed (Table 2). Interestingly, the results were dissimilar for both genes, i.e., the independent variables for which the different categories presented odd ratios (ORs) for pseudogenization statistically different from the reference were not the same for *mclx2* and *mclx3*. The only variable that had a statistically significant association with the pseudogenization for both genes was “transmissibility” (Table 2). However, the effect of this variable had a different direction in each of the genes, i.e., non-transmissible isolates were around four times more likely to carry a pseudogenized copy of *mclx2*, but around two times more likely to carry a non-pseudogenized copy of *mclx3*.

**Table 2:** Univariate ORs for *mclx*2 and *mclx*3 pseudogenization (*p* values <0.2 are highlighted).

			univariate ORs (95% CI)	
independent variables		*n*	*mclx*2 pseudogenization	*mclx*3 pseudogenization
**patient-related**				
**age**		126	**0.973 (0.942–1.006); *p* = 0.103**	1.008 (0.983–1.033); *p* = 0.553
**gender**			*p* = 0.624	***p* = 0.061**
	female	47	1.255 (0.507–3.106)	**2.202 (0.965–5.024)**
	male	79	1 (ref)	**1 (ref)**
**birth region**			*p* = 0.589	***p* = 0.005**
	Africa	23	0.679 (0.216–2.137); *p* = 0.508	**0.000 (0.000-.); *p* = 0.998**
	The Americas	12	0.385 (0.073–2.022); *p* = 0.259	**0.886 (0.158–4.974); *p* = 0.890**
	Eastern Mediterranean	23	0.288 (0.072–1.154); *p* = 0.079	**0.664 (0.154–2.874); *p* = 0.584**
	Europe	38	1 (ref)	**1 (ref)**
	South East Asia	17	0.000 (0.000-.); *p* = 0.998	**3.100 (0.873–11.007); *p* = 0.080**
	Western Pacific	13	0.000 (0.000-.); *p* = 0.999	**53.143 (5.896–478.992); *p*<0.001**
**ethnicity**			***p* = 0.002**	*p* = 0.391
	native dutch	28	**4.583 (1.738–12.088)**	0.626 (0.215–1.823)
	foreign-born	97	**1 (ref)**	1 (ref)
**house setting**			*p* = 0.787	***p* = 0.033**
	rural	81	1 (ref)	**1 (ref)**
	urban	45	0.878 (0.343–2.248)	**0.345 (0.129–0.918)**
**BCG vaccination**			*p* = 0.668	*p* = 0.751
	no	27	1.306 (0.385–4.431)	0.842 (0.291–2.433)
	yes	39	1 (ref)	1 (ref)
**co-morbidities**			*p* = 0.371	*p* = 0.414
	no or unknown	109	1 (ref)	1 (ref)
	yes	18	0.494 (0.106–2.312)	0.579 (0.156–2.148)
**alcohol or drug use**			*p* = 0.949	*p* = 0.243
	no or unknown	116	1 (ref)	1 (ref)
	yes	11	0.949 (0.192–4.707)	0.287 (0.035–2.334)
**homelessness**			*p* = 0.753	*p* = 0.978
	no or unknown	123	1 (ref)	1 (ref)
	yes	4	1.499 (0.144–14.573)	1.033 (0.104–10.310)
**microorganism-related**				
**lineage**			*p* = 0.220	-
	EAI	18	0.107 (0.013–0.860); *p* = 0.036	-
	EAm	65	1 (ref)	-
	EAs	10	0.000 (0.000-.); *p* = 0.999	-
	IO	31	0.000 (0.000-.); *p* = 0.998	1
**antibiotic resistance**			*p* = 0.659	*p* = 0.979
	none or unknown	118	1 (ref)	1 (ref)
	resistant	8	1.455 (0.275–7.696)	1.023 (0.196–5.349)
**transmissibility**			***p* = 0.003**	***p* = 0.097**
	no	49	**4.242 (1.650–10.907)**	**0.467 (0.190–1.148)**
	yes	78	**1 (ref)**	**1 (ref)**
**disease-related**				
**disease localization**			*p* = 0.573	***p* = 0.002**
	pulmonary TB	82	1 (ref)	**1 (ref)**
	extra-pulmonary TB	24	0.589 (0.156–2.222); *p* = 0.435	**5.279 (1.983–14.049); *p* = 0.001**
	pulmonary and extra-pulmonary TB	20	1.375 (0.435–4.343); *p* = 0.587	**0.788 (0.205–3.038); *p* = 0.730**

The other variable revealing an association to *mclx2* was ethnicity—being a native Dutch represented a 4.5-fold increased risk of carrying a pseudogene (Table 2). The calculation of the ORs for the different Mtb lineages did not yield significant results, as most of the isolates with the *mclx2* pseudogenized belong to the EAm lineage (with a single exception—S1 Table), and for a number of lineages the number of pseudogenizations is null. However, isolates belonging to the EAI lineage did have a decreased risk for pseudogenization when compared to isolates belonging to the EAm lineage. Among the completely sequenced and publicly available Mtb complex genomes, there was also only one strain outside the EAm lineage that had a pseudogenized *mclx2* (Table 1).

Concerning the *mclx3* four other factors besides transmissibility were shown to have a significant association with its pseudogenization: gender, birth region, house setting and localization of the TB infection (Table 2). Being a female represented a 2.2-increased risk for having a pseudogenized form of *mclx*3, whereas living in an urban area represented a decrease of this risk to 0.345. On the other hand, isolates from strictly extra-pulmonary infections had an increased OR (more than five-fold higher) for *mclx3* pseudogenization when compared to strictly pulmonary strains. Finally, birth region was strongly associated with the *mclx3* gene status, with no pseudogenes identified in isolates from patients born in Africa, a decreased OR (0.886 and 0.664) for pseudogenization in patients born in the Americas and Eastern Mediterranean, and an increased OR (3.100 and 53.143) for those born in South East Asia and Western Pacific, when compared to Europe (Table 2).

To detect confounding and/or mediation factors in the relation between the different variables with a significant association, multivariate binary logistic regression analyses were performed for the pseudogenization of *mclx2* and *mclx3* (Tables 3 and 4). These analyses included, for each case, all variables that presented a *p* below 0.200 in the univariate binary logistic regression (Table 2). These variables were organized into three different blocks (patient-related, microorganism-related and disease-related), which were sequentially added to each multivariate model.

**Table 3:** Multivariate logistic regression model for *mclx*2 pseudogenization (*p* values < 0.05 are highlighted).

		multivariate ORs (95% CI)
*mclx*2		Model 1
**patient-related**		
**age**		**0.952 (0.909–0.997); *p* = 0.038**; *B* = -0.049; *S*.*E*. = 0.024; *Wald* = 4.307
**ethnicity**	native dutch	**6.628 (1.708–25.714); *p* = 0.006**; *B* = 1.891; *S*.*E*. = 0.692; *Wald* = 7.475
	foreign-born	**1 (ref)**
**microorganism-related**		
**transmissibility**	no	1.918 (0.594–6.193); *p* = 0.276; *B* = 0.651; *S*.*E*. = 0.598; *Wald* = 1.186
	yes	1 (ref)
**Omnibus Test (chi-square/*p*)**		20.039/ *p*<0.001
**Cox & Snell R** ^**2**^		0.149
**Nagelkerke R** ^**2**^		0.242
**Hosmer and Lemeshow (chi-square/*p*)**		5.568/ *p* = 0.695
***n***		124

**Table 4:** Multivariate logistic regression models for *mclx*3 pseudogenization (*p* values < 0.05 are highlighted).

		multivariate ORs (95% CI)		
*mclx*3		Model 1	Model 2	Model 3
**patient-related**				
**gender**	female	-	-	0.532(0.149–1.893); *p* = 0.330; *B* = -0.631; *S*.*E*. = 0.648; *Wald* = 0.950
	male	-	-	1 (ref)
**house setting**	rural	-	-	1 (ref)
	urban	-	-	0.433 (0.118–1.593); *p* = 0.208; *B* = -0.837; *S*.*E*. = 0.665; *Wald* = 1.586
**birth region**		-	-	***p* = 0.009**; *Wald* = 15.307
	Africa	-	-	**0.000 (0.000-.); *p* = 0.998**; *B* = -20.087; *S*.*E*. = 8006.116; *Wald* = 0.000
	The Americas	-	-	**0.929 (0.148–5.849); *p* = 0.938**; *B* = -0.074; *S*.*E*. = 0.939; *Wald* = 0.006
	Eastern Mediterranean	-	-	**0.433 (0.077–2.446); *p* = 0.344**; *B* = -0.837; *S*.*E*. = 0.883; *Wald* = 0.897
	Europe	-	-	**1 (ref)**
	South East Asia	-	-	**3.479 (0.657–18.431); *p* = 0.143**; *B* = 1.247; *S*.*E*. = 0.851; *Wald* = 2.149
	Western Pacific	-	-	**77.372 (6.357–941.774); *p* = 0.001**; *B* = 4.349; *S*.*E*. = 1.275; *Wald* = 11.631
**microorganism-related**				
**transmissibility**	no	-	0.761 (0.280–2.070); *p* = 0.593; *B* = -0.273; *S*.*E*. = 0.510; *Wald* = 0.285	2.953 (0.648–13.458); *p* = 0.162; *B* = 1.083; *S*.*E*. = 0.774; *Wald* = 1.957
	yes	-	1 (ref)	1 (ref)
**disease-related**				
**disease localization**		***p* = 0.002**; *Wald* = 12.466	***p* = 0.009**; *Wald* = 9.447	***p* = 0.025**; *Wald* = 7.342
	pulmonary TB	**1 (ref)**	**1 (ref)**	**1 (ref)**
	extra-pulmonary TB	**5.279 (1.983–14.049); *p* = 0.001**; *B* = 1.664; *S*.*E*. = 0.499; *Wald* = 11.097	**4.702 (1.630–13.560); *p* = 0.004;** *B* = 1.548; *S*.*E*. = 0.540; *Wald* = 8.206	**9.894 (1.825–53.654); *p* = 0.008**; *B* = 2.292; *S*.*E*. = 0.863; *Wald* = 7.060
	pulmonary and extra-pulmonary TB	**0.788 (0.205–3.038); *p* = 0.730**; *B* = -0.238; *S*.*E*. = 0.688; *Wald* = 0.120	**0.769 (0.199–2.978); *p* = 0.704**; *B* = -0.262; *S*.*E*. = 0.691; *Wald* = 0.144	**3.609 (0.637–20.434); *p* = 0.147**; *B* = 1.283; *S*.*E*. = 0.885; *Wald* = 2.105
**Omnibus Test (chi-square/*p*)**		12.555/ *p* = 0.002	12.843/ *p* = 0.005	56.253/ *p*<0.001
**Cox & Snell R** ^**2**^		0.095	0.097	0.360
**Nagelkerke R** ^**2**^		0.141	0.144	0.536
**Hosmer and Lemeshow (chi-square/*p*)**		0.000/ *p* = 1.000	0.081/ *p* = 0.994	6.225/ *p* = 0.514
***n***		126		

For *mclx2*, a single model was built including the microorganism-related variable transmissibility, and the patient-related variables age and ethnicity (Table 3). Transmissibility was no longer significant upon correcting for age and ethnicity. In fact, in the multivariate model only ethnicity and age present significant associations: microorganisms isolated from native Dutch have an increased tendency to be carriers of *mclx2* pseudogenes, as do microorganisms isolated from younger people. However, it should be noticed that the *p* value for the microorganism lineage is close to the cut-off (0.200), and one of its categories actually has a *p* value of 0.036. Adding this variable to the multivariate model, both age and ethnicity lose its significance (S3 Table).

For *mclx3*, three different models were built: the first one using only the disease-related variable TB localization, the second one including the microorganism-related variable transmissibility, and the third one incorporating the patient-related variables gender, house setting and birth region (Table 4). As for transmissibility, gender and house setting, their associations to *mclx3* status were no longer significant after correcting for the other variables in the model. However, birth region remained as a significant variable, with patients born in the Western Pacific having an increased (77-fold) probability of carrying a pseudogenized form of this gene when compared to patients from Europe (Table 4). Since no pseudogenes were found among strains isolated from African patients, it was not possible to perform the mathematical computation of the OR and confidence interval for this category (and for the-Log likelihood of the model no final solution could be found). However, excluding African individuals from the sample had a negligible effect on the calculation of the parameters for the other categories/variables and on the overall significance of this model (data not shown). Finally, disease localization remains as a significant variable even after correcting for the microorganism- and patient-related variables. Clinical isolates from strictly extra-pulmonary infections have a nearly 10-fold increased probability of carrying a pseudogenized form of *mclx3* when compared to isolates from strictly pulmonary forms of the disease (Model 3, Table 4). This increased propensity for *mclx3* pseudogenized forms is maintained for isolates from disseminated (pulmonary and extra-pulmonary) infections, although in a non-significant way (Model 3, Table 4).

To avoid phylogenetic redundancy, i.e., to ensure that the observed results were due to actual relations observed in the sample and not to the relative abundance of certain genotypes, the analyses were repeated using a single representative for each VNTR and RFLP type (the excluded isolates are annotated in the S1 Table). The results were similar to the previous ones in both the univariate (S4 Table) and multivariate (S5 and S6 Tables) analyses, supporting the initial deductions.

**Fig 2:**
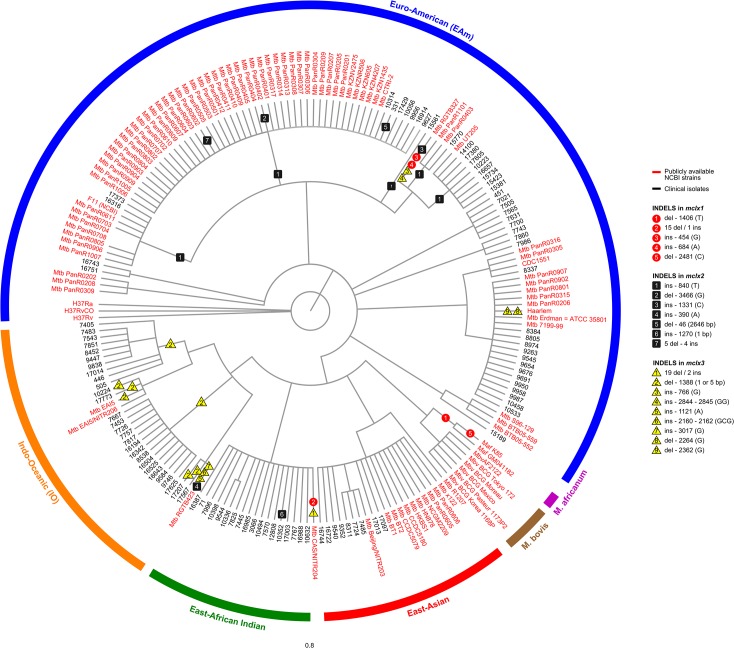
A parsimony-based phylogenetic tree depicting the distribution of pseudogenization events across the 3 *mclx* genes in both epidemiologically characterized clinical isolates (n = 100; denoted in black) and publicly available NCBI strains (n = 100; denoted in red). LSP-defined MTBC lineages/sublineages are colour-coded and indicated in the outer arc (Purple – *Mycobacterium africanum*; Brown—*Mycobacterium bovis*; blue—Euro-American lineage; red—East-Asian lineage; orange—Indo-Oceanic; green—East-African Indian lineage).

To gain some phylogenetic insight into the distribution of these pseudogenization events, a phylogenetic tree encompassing a number of genomes analysed in this article was constructed and the position of putative pseudogene-causing INDELS annotated (Fig 2). The frameshift-causing INDEL events in the different *mclx* genes are not unique in each organism, but are often found repeated across different strains/clinical isolates. On the other hand, although most pseudogenes in a given gene are concentrated in the same part of the tree, a few others appear scattered throughout the organisms, preventing a clear phylogenetic signal. Particularly, although most strains with pseudogenization events among *mclx* genes belong to Mtb, two *M*. *africanum* representatives have pseudogenized forms of the *mclx1* and there is one *M*. *bovis* BCG with a pseudogenized *mclx3* (Table 1, Figs 1 and 2). This somewhat dispersed distribution of pseudogenization events, together with the fact that different INDELs occur in different strains/clinical isolates but result in the pseudogenization of the same gene, suggest that the pseudogenization of each *mclx* may have occurred more than once in their phylogenetic history. This is consistent with a scenario where strong selective pressures are at the basis of these inactivation events, leading to the same overall result—the pseudogenization of a given *mclx*, although sometimes following different pathways (different INDELs), as opposed to a scenario of random evolution, where the pseudogenization of a given gene would have likely occurred once and dispersed throughout the lineage.

## Discussion

The analysis of the genotypic variability of the *mclx* genes revealed a scattered pattern of pseudogenization among the Mtb complex strains and clinical Mtb isolates. The occurrence of INDELs in the different *mclx* genes was not homogenous between the two panels explored, the most striking difference being that *mclx1* was pseudogenized in 7.3% of the publicly available strains compared to none in the dataset of clinical isolates. The absence of West-African 2 representatives in the latter may have contributed to this, as the pseudogenization of *mclx1* is particularly common in this lineage [20] and quite rare amongst others. Taking into account the role played by its codified protein in the macrophages' initial immune response [10], one can argue that its inactivation may be one significant aspect in *M*. *africanum* (West-African 2 members) virulence attenuation. The percentages of the pseudogenized *mclx2* and *mclx3* differed between both panels, the former being more common amongst the publicly available strains and the latter more so in the clinical isolate dataset. These differences are likely related to the degree of clustering in both panels. Whereas the clustering is limited among the studied clinical isolates, and could actually be controlled for without an impact in the main results, one cannot access such information regarding the genome-sequenced and publicly available strains. The fact that a few of them are laboratory strains, and a number of others have been likely isolated from the same given TB outbreak (such as the SUMu or the PanR collections, representing 6.7% and 39.3% of this panel, respectively), hints at the existence of higher genetic relatedness. In this context, certain genetic features may appear more common solely because they occur in an overrepresented genotype.

The existence of three *mclx* genes in each genome raises the hypothesis of functional redundancy among them. However, that would likely have as consequence a random pattern of inactivation, resulting in similar pseudogenization ratios for each gene, which is not supported by the data. Moreover, the disparate results in the logistic regression analysis in terms of significant variables buttresses the hypothesis that selective pressures and/or the clinical consequences of inactivating *mclx2* or *mclx3* are dissimilar. This is in agreement with the previously published functional analysis of the *mclx1*—Agarwall *et al*. mutated other adenylyl cyclases besides *mclx1*, namely *mclx3*, and for none of them significant effects were noticed in survival upon competing at mouse lungs, nor differences in macrophage cAMP levels compared to the wild-type, as it was for the *mclx*1, strongly suggesting that they have different functions [10].

The univariate and multivariate binary logistic regression analyses uncovered a number of statistically significant relationships that highlight the potential impact of the *mclx* genes functionality in aspects related with the microorganism biology and fitness, TB development and patients' demography. In *mclx2*, the main factor associated with pseudogenization appears to be the microorganism lineage, with all but one organism carrying a pseudogenized gene belonging to the EAm lineage among the panel of strains used for this analysis. Accordingly, in the publicly available genomes there is only one pseudogenization of *mclx2* outside the EAm lineage. Age and ethnicity might be significant factors as well, with the multivariate model showing that native Dutch patients present an approximately 6.6-fold higher risk of carrying a pseudogenized *mclx2*, and younger patients having a decreased OR for these forms. Although this could suggest some degree of adaptation it is also true that 78.6% of the native Dutch isolates actually belong to the EAm lineage, and both age and ethnicity lose significance in the multivariate model controling for lineage. As such, even if age and ethnicity do play a role in the pseudogenization of *mclx*2, this should be a rather limited one.

For *mclx3*, the results were quite dissimilar from those of *mclx2*. Significant variables in the multivariate model for *mclx3* include patient birth region and disease localization. Given the strong phylogeographical structure of Mtb lineages, the relation between the patients' birth region and *mclx3* pseudogenization can either be interpreted as a reflection of a phylogenetic signal (the effect of the microorganisms' lineage by itself cannot be evaluated for this gene, as all pseudogenes are found among strains belonging to the IO lineage in the analysed panel) or due to differences in the individuals from different regions (either genetic or socially/culturally- implemented). The relationship with disease localization remains consistently significant after correcting for all the other variables with a statistically significant signal in the univariate analysis. This suggests that *mclx3* plays a key role in the establishment of a pulmonary infection—and therefore its absence causes an adjustment of the infection to the extra-pulmonary space—or, conversely, that Mclx3 function prevents the infection from spreading.

Several risk factors for extra-pulmonary forms of TB have been addressed and characterized previously, both in what comes to the microbial influence [21–23], and also regarding host factors [24]. Concerning genetic microbial features, large INDEL polymorphisms in a phospholipase C gene, *plcD*, have been significantly associated with extra-pulmonary forms of TB when compared to strains without a *plcD* interruption [22]. On the other hand, the study of the same kind of mutations occurring in other genes from the same family, *plcA*, *plcB* and *plcC*, failed to show such a correlation [23]. This present study parallels this: *mclx3* is strongly and significantly associated with extra-pulmonary TB, but such is not the case for its paralogue *mclx2*. In what concerns host features, gender, ethnicity and HIV-status have all been found to be significant risk factors for extra-pulmonary forms of TB [24]. Whereas in this study we could not access the patients' ethnicity (concerning race/skin colour), both gender and birth region (a possible proxy) were accounted for in the multivariate model. As a precaution, the data was re-analysed integrating the *mclx3* pseudogenization status as a putative risk factor for extra-pulmonary infections (as opposed to strictly pulmonary and/or disseminated infections) and correcting for all host factors previously associated with this form of the disease: age, gender, HIV serological status, birth region and ethnicity. In accordance the pseudogenization of *mclx3* appears as an independent and highly significant risk factor for extra-pulmonary TB (S7 Table). Finally, another microbial factor that should be taken into consideration is the microbial lineage. A previous report has demonstrated that, compared to the EAs lineage, the EAm, IO and the EAI lineages are significantly associated with extra-pulmonary forms, even after correcting for relevant host factors [21]. This is particularly important in the context of this study, as almost all *mclx3* pseudogenes are found among members of the IO lineage (and actually all of them in the panel used for the regression analysis) and therefore could suggest that the *mclx3* pseudogenization is a mere phylogenetic signal. Since the occurrence of *mclx3* pseudogenes in the analysed sample is restricted to IO strains, it is not possible to correct for the lineage in the multivariate models. However, previous reports support that the EAI lineage represents a fairly similar risk for extra-pulmonary infections as the IO one [21]. Notwithstanding, in the analysed sample and among the EAI lineage there is only one case (5.6%) of strictly extra-pulmonary infection, a value that deviates significantly from the 13 cases (41.9%) observed for the IO lineage (S8 Table). Although it is not possible to completely disregard the phylogenetic hypothesis, our results suggest that the *mclx*3 pseudogenization is one of the factors that favor extra-pulmonary forms of TB, and its prevalence among the IO lineage justifies that same tendency in this lineage. Conversely, the lack of *mclx3* pseudogenes among the studied EAI could help to justify the missing tendency for extra-pulmonary infections in this particular sample.

In the context of this manuscript, pseudogene is referred to as any gene whose coding sequence has been abruptly terminated by a large or small INDEL event, leading to the accumulation of stop codons and the precocious ending of the putatively codified peptide. It does not, by any means, reflect a status of overall non-functionality. Pseudogenes can have a number of different functions in the cell. In this context, it is important to highlight the *Mycobacterium leprae*. Although *M*. *leprae* holds a large collection of pseudogenes in its genome (approximately 50%), an interestingly high number of them are actually expressed (43%), and some even vary their expression patterns upon infection or in different leprosy patients, suggesting that they can play a role in the virulence of this microorganism [25–28]. More often than not, this expression occurs from pseudogenes that have stop codons in their reading frames, as is the case of the *mclx* addressed in this study. Therefore, the *mclx* pseudogenes should be regarded as potentially functional genes, although codifying smaller proteins/peptides than their orthologues or displaying non-codifying functions. Supporting this hypothesis, one study has previously referred an over-expression of the *mclx2* after the induction of an alternative sigma factor (*sigF*) [29]. Interestingly, the SigF binding site was located within the *mclx2* coding region, resulting in a 250 residues-shorter protein. This suggests that shorter versions of at least this *mclx* may hold an important role under defined conditions.

Gene pseudogenization has been often associated with the absence of purifying selective pressures, which allow the accumulation of nucleotide substitutions and INDELs. However, in this case, the high degree of sequence conservation at a nucleotide level suggests otherwise. Frameshift-causing INDELs are sometimes the only difference in the sequences when compared with their orthologues from the reference genome Mtb H37Rv. Conversely, the *mclx* genes from the closely related *M*. *cannetti* hold a much higher degree of sequence divergence but are not pseudogenized. This suggests that the pseudogenization of the *mclx* genes is either recent and/or the result of defined selective pressures, as opposed to a longer process of genome erosion in the absence of selection. This work, by describing a family of genes selectively pseudogenized in certain isolates, reinforces the recent trend to complement immunological data with the study of bacterial evolution in order to fully understand—and control—TB.

## Supporting Information

S1 Fig A. Snapshot of a ClustalW alignment in the Geneious software calling a one base-pair insertion in codon 279 of the *mclx2* gene in a publicly available NCBI strain. **B.** Snapshot of the breseq software calling the same insertion in an epidemiologically characterized clinical isolate whose genome has been fully sequenced. Displayed are color-coded Illumina sequencing reads mapping to the H37Rv reference sequence (singled out at the top). **C.** Snapshot of a ClustalW alignment in the Geneious software calling the same insertion in an epidemiologically characterized clinical isolate in which only the *mclx* genes have been sequenced.
(PDF)

S1 Table Clinical and epidemiological characteristics of the 127 Mtb isolates analysed by binary logistic regression. (PDF)

S2 Table PCR primers and conditions for *mclx* amplification. (PDF)

S3 Table Multivariate ORs for *mclx*2 including the "microorganism lineage" as a variable. (PDF)

S4 Table Univariate ORs for *mclx*2 and *mclx*3 pseudogenization (clinical isolates with unique RFLP and VNTR). (PDF)

S5 Table Multivariate logistic regression models for *mclx*2 pseudogenization (clinical isolates with unique RFLP and VNTR). (PDF)

S6 Table Multivariate logistic regression models for *mclx*3 pseudogenization (clinical isolates with unique RFLP and VNTR). (PDF)

S7 Table Multivariate logistic regression models for extra-pulmonary TB infections. (PDF)

S8 Table Crosstabulation of Mtb lineage *vs*. local of infection. (PDF)
