# Supplementary material for: To Be or Not to Be a Pseudogene: A Molecular Epidemiological Approach to the mclx Genes and Its Impact in Tuberculosis
Source: PLoS One. 2015 Jun 2;10(6):e0128983. doi: 10.1371/journal.pone.0128983 (PMC4452763; doi:10.1371/journal.pone.0128983)
Supplement: S1 Table — (PDF) [file pone.0128983.s002.pdf]

iso	transm	year	birth_c	birth_reg	gen	age	TB_local	lineage	LSP_lineage	rx	ethn	alc_drug	homeless	h_set	BGC	mclx1	mclx2	mclx3	RFLP	VNTR	HIV	DM	malig	r_insuf	o_transp	med	co_morb
71	1	2008	Nigeria	af	1	58	3	CAS	EAI	0	1	0	0	2	1	0	0	0	0	9001110	0	0	0	0	0	0	0
331	0	2009	Senegal	af	1	24	1	LAM	EAm	0	1	0	0	2	9	0	del-1526658-2646	0	0	9003256	0	0	0	0	0	0	0
446	1	2009	Myanmar	sea	0	57	2	EAI	IO	0	1	0	0	1	9	0	0	del-2799489-1	0	9001132	0	0	0	0	0	0	0
451	0	2009	Ecuador	am	0	38	1	H	EAm	0	1	0	0	1	1	0	0	0	0	9003263	0	0	0	0	0	0	0
505	0	2009	Netherlands	eu	1	47	1	EAI	IO	0	0	0	0	1	0	0	0	del-2799489-5	0	9003246	0	0	0	0	0	0	0
1160	1	2010	Cameroon	af	0	36	2	LAM	EAm	0	1	0	0	2	9	9	0	0	0	9001822	1	0	0	0	0	0	1
3066	0	2002	Morocco	em	0	25	1	CAS	EAI	0	1	0	0	1	1	0	0	0	1181	0	0	0	0	0	0	0	0
7021	1	2001	Cameroon	af	1	30	3	HAARLEM	EAm	0	1	0	0	2	9	0	0	0	860	0	1	0	0	0	0	0	1
7405	1	2001	Somalia	em	1	18	3	EAI	IO	0	1	0	0	1	0	0	0	del-2799489-5	295123	0	0	0	0	0	0	0	0
7445	1	2002	India	sea	1	30	3	CAS	EAI	0	1	0	0	2	9	0	0	0	986	0	0	0	0	0	0	0	0
7453	1	2002	Indonesia	sea	0	25	2	EAI	IO	0	1	0	0	1	0	0	0	del-2799489-5	858	0	0	0	0	0	0	0	0
7483	1	2002	Vietnam	wp	0	24	2	EAI	IO	0	1	0	0	1	9	0	0	del-2799489-1	295017	0	0	0	0	0	0	0	0
7485	1	2002	Algeria	af	1	19	1	BEIJING	EAs	0	1	0	0	1	1	0	0	0	1151	0	0	0	0	0	0	0	0
7505	1	2002	Turkey	eu	1	24	1	H	EAm	0	1	0	0	1	1	0	0	0	653	0	0	0	0	0	0	0	0
7543	1	2002	Vietnam	wp	1	40	1	EAI	IO	1	1	0	0	1	0	0	0	del-2799489-5	1128134	0	0	0	0	0	0	0	0
7565	1	2002	Afganistan	em	1	22	1	H	EAm	0	1	0	0	1	1	0	0	0	859	0	0	0	0	0	0	0	0
7570	0	2002	Somalia	em	0	36	3	CAS	EAI	0	1	0	0	1	9	0	0	0	1134	0	0	0	0	0	0	0	0
7587	1	2002	Burma	sea	1	45	1	EAI	IO	1	1	0	0	1	1	9	0	del-2799489-5	295138	0	1	0	0	0	0	0	1
7631	1	2002	Cape Verde	af	1	19	3	LAM	EAm	0	1	0	0	2	9	0	0	0	744	0	0	0	0	0	0	0	0
7667	0	2002	Netherlands	eu	1	47	1	EAI	IO	0	0	0	0	1	0	0	0	del-2799489-5	5865	0	0	0	0	0	0	0	0
7688	1	2002	Eritrea	af	1	39	2	T	EAm	0	1	0	0	1	9	9	0	0	154	0	0	0	0	0	0	0	0
7696	1	2002	Thailand	sea	0	31	3	BEIJING	EAs	0	1	0	0	2	9	0	0	0	5891	0	0	0	0	0	0	0	0
7700	1	2002	Armenia	eu	1	39	1	H	EAm	0	1	0	0	1	0	0	0	0	1170	0	0	0	0	0	0	0	0
7724	1	2002	Afganistan	em	1	24	1	BEIJING	EAs	0	1	0	0	2	9	0	0	0	1151	0	0	0	0	0	0	0	0
7726	1	2002	Philippines	wp	0	22	1	EAI	IO	0	1	0	0	1	1	0	0	del-2799489-5	465	0	0	0	0	0	0	0	0
7743	1	9	9	9	9	999	9	LAM	EAm	9	0	0	0		9	0	0	0	744	0	0	0	0	0	0	0	0
7757	1	2002	Netherlands	eu	0	83	2	EAI	IO	0	0	0	0	1	9	0	0	del-2799489-5	549	0	0	0	1	0	0	0	1
7767	1	2002	Somalia	em	0	31	1	CAS	EAI	0	1	0	0	1	1	0	0	0	1178	0	0	0	0	0	0	0	0
7817	1	2002	Philippines	wp	0	32	2	EAI	IO	0	1	0	0	2	9	0	0	del-2799489-5	949	0	0	0	0	0	0	0	0
7825	1	2002	Pakistan	em	1	52	1	CAS	EAI	0	1	0	0	2	9	0	0	0	556	0	0	0	0	0	0	0	0
7851	1	2002	Vietnam	wp	0	26	2	EAI	IO	0	1	0	0	1	1	0	0	del-2799489-5	295017	0	0	0	0	0	0	0	0
7860	1	2002	Suriname	am	1	54	1	H	EAm	0	1	0	0	2	9	0	0	0	994	0	0	0	0	0	0	0	0
7986	1	2003	Turkey	eu	1	40	1	T	EAm	0	1	0	0	1	9	0	0	0	272	0	0	0	0	0	0	0	0
7996	0	2003	Eritrea	af	1	17	1	CAS	EAI	0	1	0	0	1	9	0	0	0	1449	0	0	0	0	0	0	0	0
8222	1	2003	Italy	eu	1	58	1	U	9	1	1	0	0	1	0	0	0	0	73	0	0	0	0	0	0	0	0
8311	0	2003	Guinea	af	1	21	1	BEIJING	EAs	0	1	0	0	2	9	0	0	0	6446	0	0	0	0	0	0	0	0
8337	1	2003	Etiopie	af	1	44	1	LAM	EAm	0	1	0	0	2	9	0	0	0	286005	0	0	0	0	0	0	0	0
8384	1	2003	Morocco	em	1	65	2	T	EAm	0	1	0	0	2	1	0	0	0	97	0	0	1	0	0	0	0	1
8452	1	2003	Vietnam	wp	1	30	1	EAI	IO	0	1	0	0	1	1	0	0	del-2799489-5	1128134	9000031	0	0	0	0	0	0	0
8538	1	2004	Indonesia	sea	1	65	2	EAI	IO	0	1	0	0	1	1	0	0	del-2799489-5	1212	9000085	0	0	0	0	0	0	0
8805	1	2004	Indonesia	sea	1	62	1	H	EAm	0	1	0	0	1	1	0	0	0	227	9000157	0	0	0	0	0	0	0
8974	1	2004	Turkey	eu	1	28	1	T	EAm	0	1	0	0	2	1	0	0	0	154	9000791	0	0	0	0	0	0	0
8989	1	2005	Kenya	af	0	31	3	LAM	EAm	0	1	0	0	2	9	0	ins-1527449-1	0	7004	9000802	1	0	0	0	0	0	1
9263	1	2005	Irak	em	1	37	2	H	EAm	0	1	0	0	1	1	0	0	0	528	9001150	0	0	0	0	0	0	0
9352	1	2005	Netherlands	eu	1	45	1	BEIJING	EAs	0	0	1	0	1	0	0	0	0	1504	9000467	0	0	0	0	0	0	0
9447	1	2006	Vietnam	wp	0	29	1	EAI	IO	0	1	0	0	1	1	0	0	del-2799489-5	1128134	9001362	0	0	0	0	0	0	0
9540	1	2006	Indonesia	sea	0	48	1	BEIJING	EAs	0	1	0	0	1	9	0	0	0	1189	9000726	0	0	0	0	0	0	0
9544	1	2006	Irak	em	1	31	1	CAS	EAI	0	1	0	0	1	9	0	0	0	870	9000289	0	0	0	0	0	0	0
9545	1	2006	Etiopie	af	1	27	1	T	EAm	1	1	0	0	1	9	0	0	0	154	9001899	1	0	0	0	0	0	1
9584	1	2006	Philippines	wp	0	50	2	EAI	IO	0	1	0	0	1	1	0	0	del-2799489-5	465	9000270	0	0	0	0	0	0	0
9627	1	2006	Morocco	em	0	28	3	LAM	EAm	0	1	0	0	1	1	0	ins-1527449-1	0	1472	9001596	0	0	0	0	0	0	0
9654	1	2006	Swaziland	af	0	35	3	H	EAm	0	1	0	0	1	1	0	0	0	227	9001257	0	0	0	0	0	0	0
9676	1	2006	Afganistan	em	0	71	2	H	EAm	0	1	0	0	1	9	0	0	0	859	9001653	0	0	0	0	0	0	0

9691	1	2006	Irak	em	0	76	1	S	EAm		0	1	0	0	1	9	0	0	0	635	9001670	0	1	0	0	0	0	1
9746	1	2006	Philippines	wp	0	26	1	EAI	IO		0	1	0	0	1	1	0	0	del-2799489-5	1510	9001746	0	0	0	0	0	0	0
9838	1	2007	Vietnam	wp	0	46	2	EAI	IO		0	1	0	0	1	1	0	0	del-2799489-5	342017	9001878	0	0	0	0	0	0	0
9950	1	2007	Morocco	em	0	71	1	H	EAm		0	1	0	0	2	9	0	0	0	1394	9002012	0	0	0	0	0	0	0
9956	1	2007	Netherlands	eu	1	46	1	LAM	EAm		0	9	0	0	1	9	0	ins-1527449-1	0	3225	9000948	0	0	0	0	0	0	0
9958	1	2007	Afganistan	em	0	66	1	H	EAm		0	1	0	0	1	9	0	0	0	262	9002021	0	1	0	0	0	0	1
9987	1	2007	Turkey	eu	1	34	1	H	EAm		0	1	0	0	2	9	0	0	0	927	9001799	0	0	0	0	0	0	0
10056	1	2007	Morocco	em	1	49	2	LAM	EAm		0	1	0	0	2	9	0	ins-1527449-1	0	6962	9000742	0	0	0	0	0	0	0
10223	1	2005	Angola	af	0	18	3	LAM	EAm		0	1	0	0	1	1	0	ins-1527449-1	0	1484	9001168	0	0	0	0	0	0	0
10224	1	2005	Netherlands	eu	0	62	1	EAI	IO		0	9	0	0	1	9	0	0	del-2799489-5	295106	9001122	0	0	0	0	0	1	0
10314	1	2008	Morocco	em	0	32	2	LAM	EAm		0	1	0	0	2	9	0	ins-1527449-1	0	6962	9000742	0	0	0	0	0	0	0
10336	1	2008	India	sea	1	28	1	CAS	EAI		0	1	0	0	2	9	0	0	0	870	9000289	0	0	0	0	0	0	0
10352	0	2008	Brazil	am	1	28	1	CAS	EAI		0	1	0	0	1	9	0	ins-1527882-1	0	8476	9002640	0	0	0	0	0	0	0
10398	1	2008	India	sea	1	24	1	CAS	EAI		0	1	0	0	2	9	0	0	0	895	9000289	0	0	0	0	0	0	0
10458	1	2008	Netherlands	eu	1	67	1	H	EAm		0	0	0	0	1	0	0	0	0	439	9002767	0	0	0	0	0	1	0
10494	1	2008	Suriname	am	0	48	2	CAS	EAI		0	1	0	0	2	9	0	0	0	870	9000289	0	0	0	0	0	0	0
10533	1	2008	Netherlands	eu	1	71	1	H	EAm		0	0	0	0	1	0	0	0	0	8000	9002846	0	0	0	0	0	0	0
10632	1	2008	Afganistan	em	1	61	1	CAS	EAI		0	1	0	0	1	1	0	0	0	884	0	0	0	0	0	0	0	0
12808	0	2004	Suriname	am	1	34	3	CAS	EAI		0	1	1	0	2	9	0	0	0	3163	9000318	0	0	0	0	0	0	0
14100	0	2007	Netherlands	eu	0	40	1	LAM	EAm		0	0	0	0	1	0	0	ins-1527449-1	0	8620	9001763	0	0	0	0	0	0	0
15107	0	2001	Algeria	af	1	45	1	U		9	0	1	1	0	2	1	0	0	0	5600	0	0	0	0	0	0	0	0
15189	0	2002	Suriname	am	1	32	1	H	EAm		0	1	1	0	2	9	0	0	0	5683	0	0	0	0	0	0	0	0
15261	0	2002	Angola	af	1	15	1	LAM	EAm		0	1	0	0	1	9	0	ins-1527449-1	0	5758	0	0	0	0	0	0	0	0
15350	0	2002	Angola	af	1	16	1	LAM	EAm		0	1	0	0	1	1	0	ins-1527449-1	0	5849	0	0	0	0	0	0	0	0
15381	0	2002	Netherlands	eu	0	30	3	LAM	EAm		0	0	0	0	1	0	0	ins-1527449-1	0	5880	0	0	0	0	0	0	0	0
15423	0	2002	Netherlands	eu	1	22	1	LAM	EAm		1	0	0	0	1	0	0	ins-1527449-1	0	1165	0	0	0	0	0	0	0	0
15424	0	2002	Netherlands	eu	0	33	1	HAARLEM	EAm		0	0	0	0	1	0	0	0	0	5925	0	0	0	0	0	0	0	0
15498	0	2002	Etiopie	af	1	15	3	X	EAm		0	1	0	0	1	9	0	0	0	6000	0	0	0	0	0	0	0	0
15561	0	2002	Netherlands	eu	0	28	1	LAM	EAm		0	0	0	0	1	9	0	ins-1527449-1	0	6066	0	0	0	0	0	0	1	0
15734	0	2003	Netherlands	eu	1	56	1	LAM	EAm		0	0	0	0	1	0	0	ins-1527449-1	0	6244	0	0	0	0	0	0	0	0
15754	0	2003	Sierra Leone	af	1	28	1	T	EAm		1	1	0	0	1	1	0	0	0	6267	0	0	0	0	0	0	0	0
15765	0	2003	Netherlands	eu	1	49	1	EAI	IO		0	0	1	1	2	0	0	0	del-2799489-5	6278	0	0	0	0	0	0	0	0
15770	0	2003	Netherlands	eu	1	41	1	LAM	EAm		0	0	1	0	2	0	0	ins-1527449-1	0	6283	0	0	0	0	0	0	0	0
15995	0	2003	Nigeria	af	0	29	1	LAM	EAm		0	1	0	1	2	9	0	0	0	6517	0	0	0	0	0	0	0	0
16194	0	2004	Netherlands	eu	0	37	3	EAI	IO		0	1	0	0	2	9	0	0	del-2799489-5	6722	9000354	0	0	0	0	0	0	0
16316	0	2007	Brazil	am	0	33	1	LAM	EAm		0	1	0	0	1	1	0	ins-1527449-1	0	8645	9002420	0	0	0	0	0	0	0
16342	1	2004	Indonesia	sea	0	30	1	EAI	IO		0	1	0	0	1	1	0	0	del-2799489-5	6875	9000605	0	0	0	0	0	0	0
16347	0	2004	Netherlands	eu	1	43	1	HAARLEM	EAm		0	0	1	0	2	0	0	0	0	6880	9000610	1	0	0	0	0	0	1
16353	0	2004	Etiopie	af	1	19	1	HAARLEM	EAm		0	1	0	0	2	9	0	0	0	6886	9000625	1	0	0	0	0	0	1
16369	1	2004	Netherlands	eu	0	83	2	H	EAm		0	0	0	0	1	0	0	0	0	6902	9000653	0	0	0	0	0	0	0
16383	0	2004	Netherlands	eu	1	34	1	T	EAm		0	0	0	0	2	0	0	0	0	6916	9000670	1	0	0	0	0	0	1
16387	0	2004	Suriname	am	1	44	1	CAS	EAI		0	1	1	0	2	9	0	0	0	6920	9000676	0	0	0	0	0	0	0
16443	0	2004	Suriname	am	1	60	1	EAI	IO		0	1	0	0	2	9	0	0	del-2799489-5	6981	9000055	1	0	0	0	0	0	1
16460	0	2004	Angola	af	1	37	3	LAM	EAm		0	1	0	1	1	1	0	ins-1527449-1	0	6999	9000796	1	0	0	0	0	0	1
16504	1	2005	Indonesia	sea	0	30	2	EAI	IO		0	1	0	0	2	9	0	0	del-2799489-5	7044	9000817	0	0	0	0	0	0	0
16525	1	2005	Suriname	am	1	55	3	EAI	IO		0	1	0	0	2	9	0	0	del-2799489-5	7066	9000055	0	0	0	0	0	0	0
16565	0	2005	Afganistan	em	1	22	1	NEW-1		9	0	1	1	0	1	1	0	0	0	7110	9000953	0	0	0	0	0	0	0
16643	1	2005	Philippines	wp	0	27	2	EAI	IO		0	1	0	0	1	1	0	0	del-2799489-1	7191	9000817	0	0	0	0	0	0	0
16657	0	2005	Netherlands	eu	1	49	1	LAM	EAm		0	0	0	0	1	1	0	ins-1527449-1	0	7207	9001083	0	0	0	0	0	0	0
16722	0	2005	Suriname	am	1	47	1	BEIJING	EAs		0	1	0	0	2	9	0	0	0	7274	9001183	1	0	0	0	0	0	1
16743	0	2005	Netherlands	eu	0	35	3	T	EAm		1	0	1	0	2	0	0	0	0	7295	9001219	1	0	0	0	0	0	1
16744	1	2005	Vietnam	wp	1	40	1	BEIJING	EAs		0	1	0	0	1	1	0	0	0	7296	9000726	0	0	0	0	0	0	0
16751	0	2005	Eritrea	af	1	27	1	HAARLEM	EAm		0	1	0	0	1	9	0	0	0	7303	9001232	1	0	0	0	0	0	1
16764	1	2005	Indonesia	sea	0	47	2	H	EAm		0	1	0	0	1	0	0	0	0	3821	9000653	0	0	0	0	0	0	0

16914	1	2006	Turkey	eu	1	30	1	LAM	EAm	0	1	0	0	1	1	0	ins-1527449-1	0	8027	9000209	0	0	0	0	0	0	0
16928	0	2006	Dominican I	am	0	38	1	H	EAm	0	1	0	0	1	0	0	0	0	8050	9001489	0	0	0	0	0	0	0
16985	0	2006	Netherlands	eu	1	27	1	CAS	EAI	0	1	0	0	1	9	0	0	0	8100	9001571	0	0	0	0	0	0	0
16988	1	2006	India	sea	0	29	1	CAS	EAI	0	1	0	0	2	1	0	0	0	8107	9001576	0	0	0	0	0	0	0
17003	0	2006	Somalia	em	1	24	3	CAS	EAI	0	1	0	1	1	9	0	0	0	8115	9000603	0	0	0	0	0	0	0
17010	1	2006	Netherlands	eu	1	68	1	H	EAm	0	0	0	0	1	9	0	0	0	8128	9000653	0	0	0	0	0	0	0
17013	1	2006	Indonesia	sea	1	78	1	BEIJING	EAs	0	1	0	0	1	9	0	0	0	3700	9000784	0	1	0	0	0	0	1
17014	0	2006	Netherlands	eu	1	59	1	EAI	IO	0	0	0	0	1	0	0	0	del-2799489-5	8131	9001615	0	0	0	0	0	0	0
17097	1	2006	Indonesia	sea	1	26	1	BEIJING	EAs	0	1	0	0	1	9	0	0	0	8239	9001767	0	0	0	0	0	0	0
17207	0	2007	Somalia	em	1	25	1	EAI	IO	0	1	0	0	1	0	0	0	del-2799489-5	8515	9001914	0	0	0	0	0	0	0
17248	0	2007	Netherlands	eu	0	41	1	H	EAm	0	0	0	0	2	1	0	0	0	8394	9001985	0	0	0	0	0	0	0
17373	0	2007	Netherlands	eu	1	37	1	LAM	EAm	0	0	0	0	2	9	0	ins-1527449-1	0	8522	9002240	0	0	0	0	0	0	0
17380	0	2007	Netherlands	eu	0	36	1	LAM	EAm	0	0	0	0	2	9	0	ins-1527449-1	0	8523	9002252	0	0	0	0	0	0	0
17429	1	2007	Netherlands	eu	0	57	2	LAM	EAm	0	0	0	0	1	0	0	ins-1527449-1	0	8563	9000209	0	0	0	0	0	0	0
17517	1	2005	Morocco	em	1	55	3	S	EAm	0	1	0	0	2	9	0	0	0	7322	9000081	0	0	0	0	0	0	0
17567	1	2008	Somalia	em	1	51	2	EAI	IO	0	1	0	0	1	9	0	0	del-2799489-5	8752	9002492	0	0	0	0	0	0	0
17605	0	2008	Netherlands	eu	1	40	1	LAM	EAm	1	0	1	0	2	9	0	ins-1527449-1	0	8732	9002541	0	0	0	0	0	0	0
17625	1	2008	Philippines	wp	1	23	1	EAI	IO	0	1	0	0	1	1	0	0	del-2799489-5	8749	9000817	0	0	0	0	0	0	0
17667	0	2008	Netherlands	eu	1	20	1	LAM	EAm	0	0	0	0	1	0	0	0	0	8799	9002627	0	0	0	0	0	0	0
17773	0	2008	Sri Lanka	sea	1	37	2	EAI	IO	0	1	0	0	1	1	0	0	del-2799489-1	9067	9000589	0	0	0	0	0	0	0

iso= isolate anonymous code; transm=transmissibility, 1=yes, 0=no; birth_c=birth country; birth_r=birth region according to the World Health Organization regions, af= Africa, am= The Americas, em= Eastern Mediterranean, eu= Europe, sea= South East Asia, wp= Western Pacific; gen=gender, 0=female, 1=male; TB_local=localization of the TB infection, 1=pulmonar, 2=extra-pulmonar, 3=pulmonar and extra-pulmonar; lineage=lineage according to spoligotype; LPS_lineage=lineage according to large sequence polymorphisms, EAs=East-Asian Lineage, EAI=East-African-Indian lineage, IO=Indo-Oceanic lineage, EAm=Euro-American lineage, 9=unknown/unclassified; rx= antibiotic resistance, 0=normally susceptible or unknown, 1-any kind of resistance; ethn=ethnicity, 0=native dutch, 1=foreign-born (including second generation); alc_drug= alchool or/and drug addition, 0=no addition or unknown, 1=addition to alcohol and/or drugs; homeless=homelessness, 0=no or unknown, 1=yes; h_set=house setting, 1=rural, 2=urban; BGC=BGC vaccination, 0=no, 1=yes, 9=unknown; mclx1, 2 or 3=gene status, 0=functional, 9=unknown; pseudogenes=indicated by the event (ins or del), its location using as reference the Mtb H37Rv genome, and the number of nucleotides affected; RFLP=restriction fragment length polymorphism cluster; VNTR=variable number of tandem repeats cluster; HIV=co-infection with HIV, 0=no, 1=yes; DM=diabetes mellitus, 0=no or unknown, 1=yes; malig=presence of malignancies, 0=no or unknown, 1=yes; r_insuf=renal insuficiency, 0=no or unknown, 1=yes; o_transp=recent organ transplantation, 0=no or unknown, 1=yes;med=intake of medication, 0=no or unknown, 1=yes; co_morb=co-morbidities, 0=no or unknown, 1-at least one of the 5 considered co-morbidities is present (HIV, DM, malig, r_insuf, and o_transp). Isolates that are redundant (ie., belong to the same RFLP and/or the same VNTR as another isolate in the sample), and that were removed from the analysis for the supplementary tables 3 to 5, are highlighted in grey.
