# Supplementary material for: To Be or Not to Be a Pseudogene: A Molecular Epidemiological Approach to the mclx Genes and Its Impact in Tuberculosis
Source: PLoS One. 2015 Jun 2;10(6):e0128983. doi: 10.1371/journal.pone.0128983 (PMC4452763; doi:10.1371/journal.pone.0128983)
Supplement: S2 Table — (PDF) [file pone.0128983.s003.pdf]

Supporting table 2

gene	primers' sequence		localization in the genome (nt)
mclx1 (463411-466668)	1.1Fw	CGGTGGCGTCGCTTCGACAT	463328-463347
	1.3Rev	CACCGAGGCCCACAGCGTC	464700-464682
	1.2 Fw	GGCCGGACTTTCGCCTCACC	464486-464505
	1.2Rev	CTGGTAGGCGAGCGCGAAGG	465780-465761
	1.3Fw	CCCAAGAGGCACGCGAGCTG	465569-465588
	1.1Rev	CCGTCCCCGAACGCCAATCA	466695-466676
mclx2 (1526612-1530091)	2.1Fw	CCAGCGGTTTCCTACGGGCG	1526542-1526561
	2.3Rev	CGCCGGCAGATCTCGCTCAC	1527960-1527941
	2.2Fw	TGGGTGCCTGCCCGGAGTTA	1527762-1527781
	2.2Rev	TCTGCGCCAGGCAGGCAAAC	1529081-1529062
	2.3Fw	CCGAGGCGATCGAGCTGGC	1528881-1528899
	2.1Rev	GCGACAACGCGCAGAAGAGC	1530170-1530151
mclx3 (2797467-2800880)	3.1Fw	ACCTTTGGTCGCTGGCTGGC	2797439-2797458
	3.3Rev	GGATCTGGCGCGACCGTGG	2798733-2798715
	3.2Fw	CCCTGCCAGAGATTCGCCGC	2798497-2798516
	3.2Rev	GCGGCTCTGATCGTCGCGTT	2799830-2799811
	3.3Fw	CAGGGCGAGGTTGTCGGCAG	2799633-2799652
	3.1Rev	CACGGGCACTGTAGGTCCGC	2800950-2800931
