# Supplementary material for: To Be or Not to Be a Pseudogene: A Molecular Epidemiological Approach to the mclx Genes and Its Impact in Tuberculosis
Source: PLoS One. 2015 Jun 2;10(6):e0128983. doi: 10.1371/journal.pone.0128983 (PMC4452763; doi:10.1371/journal.pone.0128983)
Supplement: S3 Table — (PDF) [file pone.0128983.s004.pdf]

Supporting Table 3

***mclx*#2**			**multivariate ORs (95% CI)**
			Model 2
**patient-related**	**age**		0.958 (0.912-1.007)<br>*p*=0.089<br>*B*=-0.043; *S.E.*=0.025<br>*Wald*=2.897
	**ethnicity**	native dutch	3.702 (0.811-16.898)<br>*p*=0.091<br>*B*=1.309; *S.E.*=0.775<br>*Wald*=2.854
		foreign-born	1 (ref)
**microorganism-related**	**transmissibility**	no	1.754 (0.439-7.000)<br>*p*=0.426<br>*B*=0.562; *S.E.*=0.706<br>*Wald*=0.632
		yes	1 (ref)
	**lineage**		*p*=0.357<br>*Wald*= 3.237
		EAI	0.136 (0.015-1.196)<br>*p*=0.072<br>*B*=-1.998; *S.E.*=1.111<br>*Wald*=3.237
		EAm	1 (ref)
		EAs	p=0.999<br>0.000 (0.000-.)<br>B=-20.230; S.E.=12299.315<br>Wald=0.000
		IO	*p*=0.998<br>0.000 (0.000-.)<br>*B*=-20.251; *S.E.*=7120.660<br>*Wald*=0.000
**Omnibus Test (chi-square/*p*)**			40.620/ *p*<0.001
**Cox & Snell R^2**			0.285
**Nagelkerke R^2**			0.459
**Hosmer and Lemeshow (chi-square/*p*)**			0.792/ *p*=0.999
n			121
