# Supplementary material for: To Be or Not to Be a Pseudogene: A Molecular Epidemiological Approach to the mclx Genes and Its Impact in Tuberculosis
Source: PLoS One. 2015 Jun 2;10(6):e0128983. doi: 10.1371/journal.pone.0128983 (PMC4452763; doi:10.1371/journal.pone.0128983)
Supplement: S4 Table — (PDF) [file pone.0128983.s005.pdf]

Supporting table 4

independent variables			n	univariate ORs (95% CI)	
				mclx#2 pseudogenization	*mclx*#3 pseudogenization
patient-related	**age**		105	.970 (0.937-1.004)<br>*p*=0.080	1.016 (0.988-1.045)<br>*p*=0.264
	**gender**			*p*=0.463	*p*=0.178
		female	36	1.436 (0.546-3.773)	1.895 (0.747-4.809)
		male	69	1 (ref)	1 (ref)
	**birth region**			*p*=0.558	*p*=0.020
		Africa	19	0.885 (0.268-2.916)<br>*p*=0.840	.000 (.000-.)<br>*p*=0.998
		The Americas	10	0.479 (0.088-2.621)<br>*p*=0.396	0.444 (0.048-4.116)<br>*p*=0.475
		Eastern Mediterranean	20	0.213 (0.042-1.075)<br>*p*=0.061	0.706 (0.161-3.103)<br>*p*=0.645
		Europe	35	1 (ref)	1 (ref)
		South East Asia	13	0.000 (0.000-.)<br>*p*=0.999	3.429 (0.872-13.483)<br>*p*=0.078
		Western Pacific	8	0.000 (0.000-.)<br>*p*=0.999	28.000 (2.942-266.467)<br>*p*=0.004
	**ethnicity**			*p*=0.003	*p*=0.602
		native dutch	27	4.606 (1.669-12.714)	0.745 (0.247-2.250)
		foreign-born	77	1 (ref)	1 (ref)
	**house setting**			*p*=0.632	*p*=0.030
		rural	67	1 (ref)	1 (ref)
		urban	38	0.783 (0.288-2.131)	0.276 (0.087-0.883)
	**BCG vaccination**			*p*=0.721	*p*=0.873
		no	25	1.263 (0.351-4.551)	1.098 (0.349-3.458)
		yes	30	1 (ref)	1 (ref)
	**co-morbidities**			*p*=0.328	*p*=0.255
		no or unknown	89	1 (ref)	1 (ref)
		yes	17	0.460 (0.097-2.183)	0.406 (0.086-1.917)
	**alcohol or drug use**			*p*=0.824	*p*=0.280
		no or unknown	95	1 (ref)	1 (ref)
		yes	11	0.833 (0.167-4.167)	0.313 (0.038-2.578)
	**homelessness**			*p*=0.831	*p*=0.909
		no or unknown	102	1 (ref)	1 (ref)
		yes	4	1.286 (0.127-12.998)	1.145 (0.114-11.538)
microbe-related	**lineage**			*p*=0.267	-
		EAI	15	0.119 (0.015-0.972)<br>*p*=0.047	-
		EAm	56	1 (ref)	-
		EAs	8	0.000 (0.000-.)<br>*p*=0.999	-
		IO	24	0.000 (0.000-.)<br>*p*=0.998	1
	**antibiotic resistance**			*p*=0.770	*p*=0.881
		none or unknown	97	1 (ref)	1 (ref)
		resistant	8	1.283 (0.241-6.846)	1.136 (0.214-6.033)
	**transmissibility**			*p*=0.006	*p*=0.076
		no	48	4.333 (1.537-12.217)	0.412 (0.154-1.098)
		yes	58	1 (ref)	1 (ref)
disease-related	**local of infection**			*p*=0.559	*p*=0.001
		pulmonary TB	71	1 (ref)	1 (ref)
		extra-pulmonary TB	16	0.533 (0.109-2.609)<br>*p*=0.438	9.091 (2.741-30.153)<br>*p*<0.001
		pulmonary and extra-pulmonary TB	18	1.436 (0.442-4.665)<br>*p*=0.547	1.091 (0.270-4.408)<br>*p*=0.903
