# Supplementary material for: To Be or Not to Be a Pseudogene: A Molecular Epidemiological Approach to the mclx Genes and Its Impact in Tuberculosis
Source: PLoS One. 2015 Jun 2;10(6):e0128983. doi: 10.1371/journal.pone.0128983 (PMC4452763; doi:10.1371/journal.pone.0128983)
Supplement: S5 Table — (PDF) [file pone.0128983.s006.pdf]

Supporting table 5

***mclx*#2**			**multivariate ORs (95% CI)**
			Model 1
**patient-related**	**age**		0.950 (0.903-0.999)<br>*p*=0.047<br>*B*=-0.051; *S.E.*=0.026<br>*Wald*=3.940
	**ethnicity**	native dutch	6.845 (1.704-27.502)<br>*p*=0.007<br>*B*=1.924; *S.E.*=0.710<br>*Wald*=7.349
		foreign-born	1 (ref)
**microbe-related**	**transmissibility**	no	1.790 (0.485-6.606)<br>*p*=0.382<br>*B*=0.582; *S.E.*=0.666<br>*Wald*=0.764
		yes	1 (ref)
**Omnibus Test (chi-square/*p*)**			18.640/ p<0.001
**Cox & Snell R^2**			0.166
**Nagelkerke R^2**			0.260
**Hosmer and Lemeshow (chi-square/*p*)**			5.027/ *p*=0.755
**n**			103
