# Supplementary material for: To Be or Not to Be a Pseudogene: A Molecular Epidemiological Approach to the mclx Genes and Its Impact in Tuberculosis
Source: PLoS One. 2015 Jun 2;10(6):e0128983. doi: 10.1371/journal.pone.0128983 (PMC4452763; doi:10.1371/journal.pone.0128983)
Supplement: S6 Table — (PDF) [file pone.0128983.s007.pdf]

Supporting table 6

mclx#3			multivariate ORs (95% CI)		
			Model 1	Model 2	Model 3
**patient-related**	**gender**	female	-	-	0.632 (0.162-2.471)<br>*p*=0.510<br>*B*=-0.458; *S.E.*=0.695<br>*Wald*=0.434
		male	-	-	1 (ref)
	**house setting**	rural	-	-	1 (ref)
		urban	-	-	0.287(0.061-1.352)<br>*p*=0.114<br>*B*=-1.248; *S.E.*=0.791<br>*Wald*=2.492
	**birth region**		-	-	*p*=0.062<br>*Wald*=10.505
		Africa	-	-	0.000 (0.000-.)<br>*p*=0.998<br>*B*=-20.135;<br>*S.E.*=8708.272<br>*Wald*=0.000
		The Americas	-	-	0.547 (0.053-5.636)<br>*p*=0.612<br>*B*=-0.604; *S.E.*=1.190<br>*Wald*=0.257
		Eastern Mediterranean	-	-	0.320 (0.049-2.087)<br>*p*=0.233<br>*B*=-1.141; *S.E.*=0.957<br>*Wald*=1.420
		Europe	-	-	1 (ref)
		South East Asia	-	-	3.025 (0.470-19.483)<br>*p*=0.244<br>*B*=1.107; *S.E.*=0.950<br>*Wald*=1.357
		Western Pacific	-	-	24.851 (1.933-319.528)<br>*p*=0.014<br>*B*=3.213; *S.E.*=1.303<br>*Wald*=6.080
**microbe-related**	**transmissibility**	no	-	0.728 (0.241-2.199)<br>*p*=0.573<br>*B*=-0.318; *S.E.*=0.564<br>*Wald*=0.317	2.079 (0.421-10.259)<br>*p*=0.369<br>*B*=0.732; *S.E.*=0.815<br>*Wald*=0.807
		yes	-	1 (ref)	1 (ref)
**disease-related**	**local of infection**		*p*=0.001<br>*Wald*= 13.647	*p*=0.006<br>*Wald*=10.383	*p*=0.025<br>*Wald*=7.345
		pulmonary TB	1 (ref)	1 (ref)	1 (ref)
		extra-pulmonary TB	9.091 (2.741-30.153)<br>*p*<0.001<br>*B*=2.207; *S.E.*=0.612<br>*Wald*=13.018	7.860 (2.163-28.569)<br>*p*=0.002<br>*B*=2.062; *S.E.*=0.658<br>*Wald*=9.806	13.464(1.951-92.939)<br>*p*=0.008<br>*B*=2.600; *S.E.*=0.986<br>*Wald*=6.958
		pulmonary and extra-pulmonary TB	1.091 (0.270-4.408)<br>*p*=0.903<br>*B*=0.087; *S.E.*=0.712<br>*Wald*=0.015	1.056 (0.259-4.294)<br>*p*=0.940<br>*B*=0.054; *S.E.*= 0.716<br>*Wald*=0.006	4.571 (0.691-30.246)<br>*p*= 0.115<br>*B*=1.520; *S.E.*=0.964<br>*Wald*=2.484
**Omnibus Test (chi-square/*p*)**			14,269/ *p*=0.001	14.587/ *p*=0.002	42.240/ *p*<0.001
**Cox & Snell R^2**			0.127	0.130	0.331
**Nagelkerke R^2**			0.193	0.197	0.503
**Hosmer and Lemeshow (chi-square/*p*)**			0.000/ *p*=1.000	0.301/ *p*=0.960	6.527/ *p*=0.480
**n**			105		
