# Supplementary material for: To Be or Not to Be a Pseudogene: A Molecular Epidemiological Approach to the mclx Genes and Its Impact in Tuberculosis
Source: PLoS One. 2015 Jun 2;10(6):e0128983. doi: 10.1371/journal.pone.0128983 (PMC4452763; doi:10.1371/journal.pone.0128983)
Supplement: S7 Table — (PDF) [file pone.0128983.s008.pdf]

Supporting Table 7

independent variables		n	univariate ORs (95% CI) exclusively extrapulmonary (vs. pulmonary and disseminated)	univariate ORs (95% CI) extrapulmonary or both (vs. exclusivetly pulmonary)
**age**		124	1.059 (1.019-1.102)<br>*p*=0.004<br>*B*=0.058; *S.E.*= 0.020<br>*Wald*= 8.420	1.020 (0.990-1.051)<br>*p*=0.186<br>*B*=0.020; *S.E.*=0.015<br>*Wald*=1.749
**gender**	female	46	6.575 (1.896-22.802)<br>*p*=0.003<br>*B*=1.883; *S.E.*=0.634<br>*Wald*= 8.810	5.708 (2.216-14.701)<br>*p*<0.001<br>*B*=1.742; *S.E.*=0.483<br>*Wald*=13.021
	male	78	1 (ref)	1 (ref)
**birth region**			*p*=0.900<br>*Wald*=1.608	*p*=0.179<br>*Wald*=7.610
	Africa	23	6.883×10^8 (0.000-.)<br>*p*=0.999<br>*B*=20.350; *S.E.*=11556.217<br>*Wald*=0.000	11.220 (0.710-177.347)<br>*p*=0.086<br>*B*=2.418; *S.E.*=1.408<br>*Wald*= 2.947
	The Americas	12	1.307×10^8 (0.000-.)<br>*p*=0.999<br>*B*=18.689; *S.E.*=11556.217<br>*Wald*=0.000	1.874 (0.104-33.760)<br>*p*=0.670<br>*B*=0.628; *S.E.*=1.475<br>*Wald*=0.181
	Eastern Mediterranean	23	5.254×10^8 (0.000-.)<br>*p*=0.999<br>*B*=20.080; *S.E.*= 11556.217<br>*Wald*=0.000	6.247 (0.429-90.932)<br>*p*=0.180<br>*B*=1.832; *S.E.*=1.366<br>*Wald*= 1.798
	Europe	36	1 (ref)	1 (ref)
	South East Asia	17	4.683×10^8 (0.000-.)<br>*p*=0.999<br>*B*=19.965; *S.E.*= 11556.217<br>*Wald*=0.000	3.314 (0.213-51.496)<br>*p*=0.392<br>*B*=1.198; *S.E.*=1.400<br>*Wald*=0.733
	Western Pacific	13	3.313×10^8 (0.000-.)<br>*p*=0.999<br>*B*=19.619; *S.E.*=11556.217<br>*Wald*=0.000	1.077 (0.058-19.930)<br>*p*=0.960<br>*B*=0.075; *S.E.*=1.489<br>*Wald*=0.003
**ethnicity**	native dutch	27	9.348×10^7 (0.000-.)<br>*p*=0.999<br>*B*=18.353; *S.E.*=11556.217<br>*Wald*=0.000	0.995 (0.064-15.571)<br>*p*=0.997<br>*B*=-0.005; *S.E.*=1.403<br>*Wald*=0.000
	foreign-born	97	1 (ref)	1 (ref)
**HIV**	negative	111	1 (ref)	1 (ref)
	positive	13	0.497 (0.044-5.627)<br>*p*=0.572<br>B=-0.700; S.E.= 1.239<br>Wald=0.319	1.195 (0.279-5.114)<br>*p*=0.810<br>B=0.178; S.E.=0.742<br>Wald=0.058
***mclx*3 status**	pseudogene	30	8.259 (1.674-40.761)<br>*p*=0.010<br>*B*=2.111; *S.E.*=0.814<br>*Wald*=6.720	4.994 (1.430-17.436)<br>*p*=0.012<br>*B*=1.608; *S.E.*=0.638<br>*Wald*=6.354
	functional	94	1 (ref)	1 (ref)
**Omnibus Test (chi-square/*p*)**			41.614/ *p*<0.001	33.355/ *p*<0.001
**Cox & Snell R^2**			0.285	0.236
**Nagelkerke R^2**			0.456	0.324
**Hosmer and Lemeshow (chi-square/*p*)**			5.289/ *p*=0.726	5.420/ *p*=0.712
**n**			124	
