# Supplementary material for: To Be or Not to Be a Pseudogene: A Molecular Epidemiological Approach to the mclx Genes and Its Impact in Tuberculosis
Source: PLoS One. 2015 Jun 2;10(6):e0128983. doi: 10.1371/journal.pone.0128983 (PMC4452763; doi:10.1371/journal.pone.0128983)
Supplement: S8 Table — (PDF) [file pone.0128983.s009.pdf]

Supporting Table 8

			Lineage				Total
			EAI	EAm	EAs	IO	
local of infection	pumonary TB	Count	12_a	43_a	9_a	15_a	79
		% within Lineage	66,7%	67,2%	90,0%	48,4%	64,2%
	extra-pulmonary TB	Count	1_a	10_a	$0_{a, b}$	13_b	24
		% within Lineage	5,6%	15,6%	0,0%	41,9%	19,5%
	pulmonary+extra-pulmonary TB	Count	5_a	11_a	1_a	3_a	20
		% within Lineage	27,8%	17,2%	10,0%	9,7%	16,3%
Total		Count	18	64	10	31	123
		% within Lineage	100,0%	100,0%	100,0%	100,0%	100,0%

Each subscript letter denotes a subset of LSP_lineage categories whose column proportions do not differ significantly from each other at the 0.05 level (collumn proportions compared by the z-test with p-values adjusted by the Bonferroni method).
