# Supplementary material for: To Be or Not to Be a Pseudogene: A Molecular Epidemiological Approach to the mclx Genes and Its Impact in Tuberculosis
Source: PLoS One. 2015 Jun 2;10(6):e0128983. doi: 10.1371/journal.pone.0128983 (PMC4452763; doi:10.1371/journal.pone.0128983)
Supplement: S1 Fig — B. Snapshot of the breseq software calling the same insertion in an epidemiologically characterized clinical isolate whose genome has been fully sequenced. Displayed are color-coded Illumina sequencing reads mapping to the H37Rv reference sequence (singled out at the top). C. Snapshot of a ClustalW alignment in the Geneious software calling the same insertion in an epidemiologically characterized clinical isolate in which only the mclx genes have been sequenced. (PDF) [file pone.0128983.s001.pdf]

A

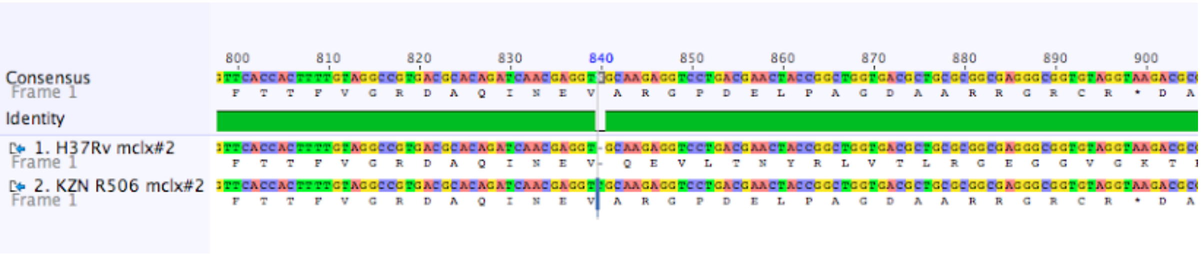

800
810
820
830
840
850
860
870
880
890
900
Consensus
Frame 1
Identity
1. H37Rv mclx#2
Frame 1
2. KZN R506 mclx#2
Frame 1

B

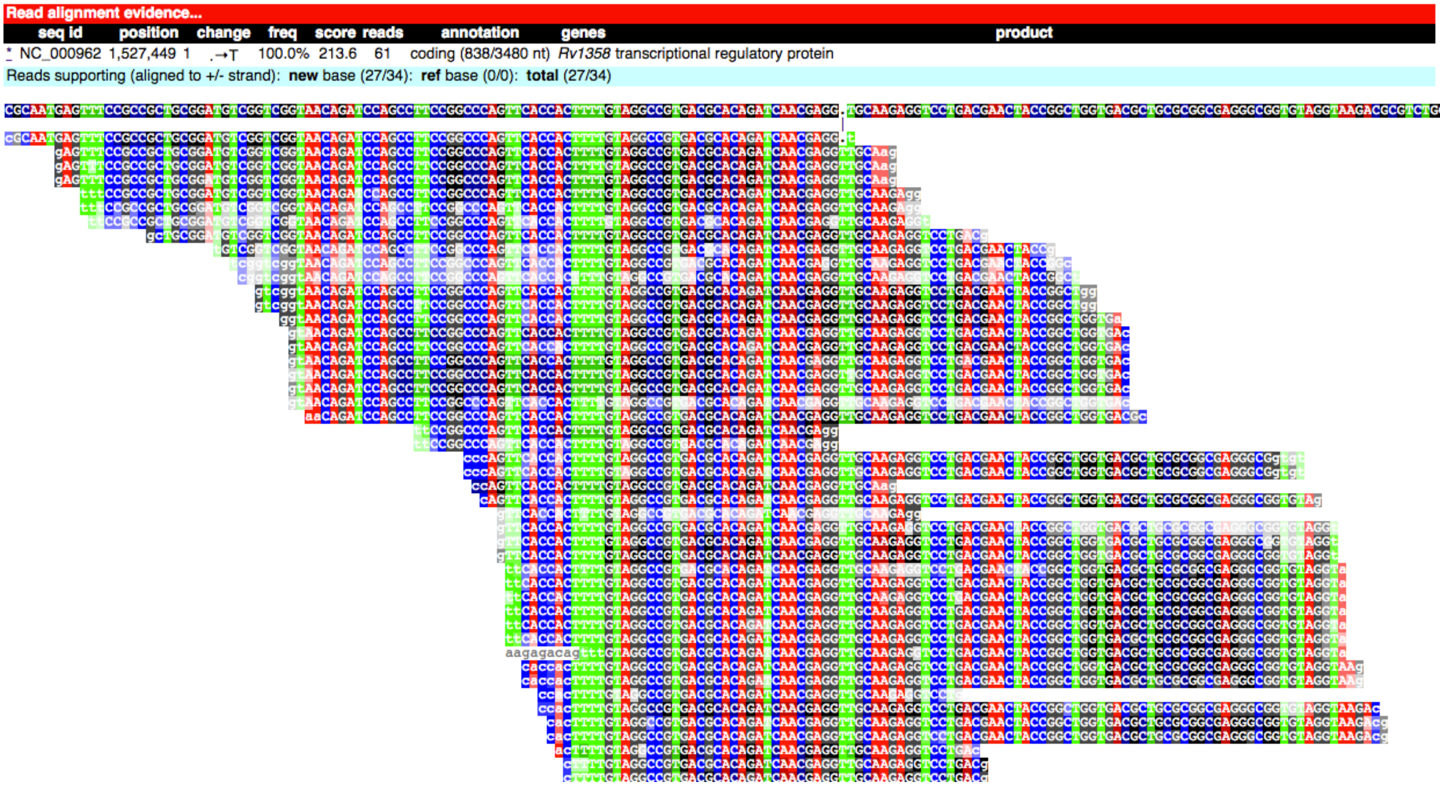

Read alignment evidence...
seq id | position | change | freq | score | reads | annotation | genes | product
* NC_000962 1,527,449 1 .→T 100.0% 213.6 61 coding (838/3480 nt) Rv1358 transcriptional regulatory protein
Reads supporting (aligned to +/- strand): new base (27/34): ref base (0/0): total (27/34)

C

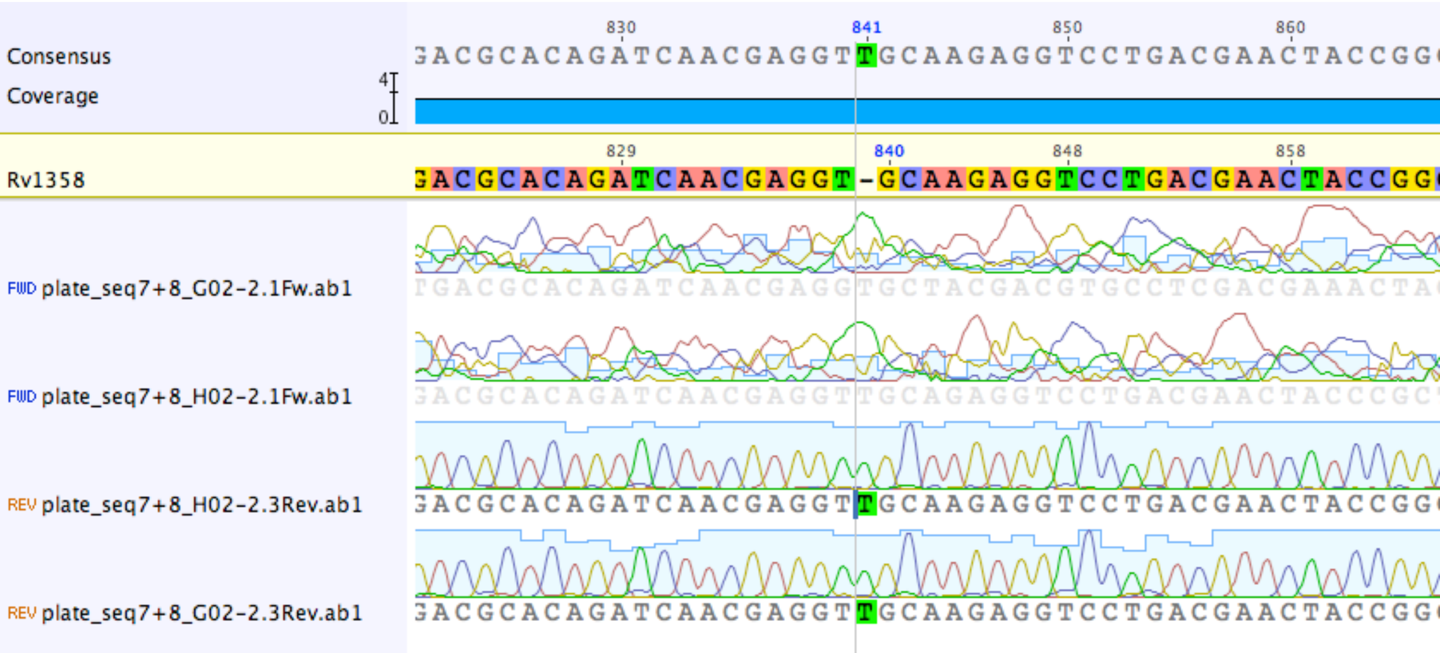

830
841
850
860
Consensus
GACGCACAGATCAACGAGGTTGCAAGAGGTCCTGACGAACTACCGG
Coverage
4
0
829
840
848
858
Rv1358
GACGCACAGATCAACGAGGT-GCAAGAGGTCCTGACGAACTACCGG
FWD plate_seq7+8_G02-2.1Fw.ab1
FWD plate_seq7+8_H02-2.1Fw.ab1
REV plate_seq7+8_H02-2.3Rev.ab1
GACGCACAGATCAACGAGGTTGCAAGAGGTCCTGACGAACTACCGG
REV plate_seq7+8_G02-2.3Rev.ab1
GACGCACAGATCAACGAGGTTGCAAGAGGTCCTGACGAACTACCGG
